# JMJD3 modulates benign prostatic hyperplasia through epigenetic regulation of TGF-β1

**DOI:** 10.1038/s41419-026-08967-9

**Published:** 2026-06-05

**Authors:** Bo Chen, Jinze Li, Yin Huang, Xinyang Liao, Mingjing He, Zeyu Chen, Jia You, Jie Chen, Qiyou Wu, Jinbao Wang, Jinjiang Jiang, Jingxing Bai, Biao Ran, Chunlan Chen, Shu Ning, Dehong Cao, Qiang Wei, Liangren Liu

**Affiliations:** 1 Department of Urology, West China Hospital, Sichuan University, Chengdu, China; 2 Institute of Urology, West China Hospital, Sichuan University, Chengdu, China; 3 Department of Urology, People’s Hospital of Deyang City, Affiliated to Chengdu University of Traditional Chinese Medicine, Deyang, China; 4 Department of Urological Oncology, Sichuan Cancer Hospital, Chengdu, China; 5 Ningbo Clinical Pathological Diagnosis Center, Ningbo, China; 6 Department of Urologic Surgery, University of California, Davis, CA USA

**Keywords:** Prostatic diseases, Chronic inflammation

## Abstract

Benign prostatic hyperplasia (BPH) is a common chronic disease among elderly men, and its etiology remains unclear. This study aimed to investigate the expression, biological function, and underlying mechanism of JMJD3 in BPH. Here, we identified that JMJD3 is a crucial driver of BPH progression. In this work, both scRNA and bulk sequencing data revealed that JMJD3 is upregulated in BPH, which was further validated in prostate tissues from BPH patients. Functionally, Overexpressing JMJD3 could promote cell proliferation and cell cycles in WPMY-1 and RWPE-1 cells, respectively. Whereas, blocking JMJD3 with GSK-J4 or siRNA may induce cell apoptosis, and inhibit cell proliferation and cell cycles in BPH-1 cells. Furthermore, inhibition of JMJD3 with GSK-J4 could alleviate both testosterone and lipopolysaccharide (LPS) induced BPH in vivo. Mechanistically, JMJD3 demethylates H3K27me3 at the promoter of TGF-β1 to promote its expression and then promotes disease progression in BPH. In summary, our findings illustrate JMJD3’s contribution to BPH progression and offer a potential JMJD3-targeted therapy for BPH.

**Figure:**
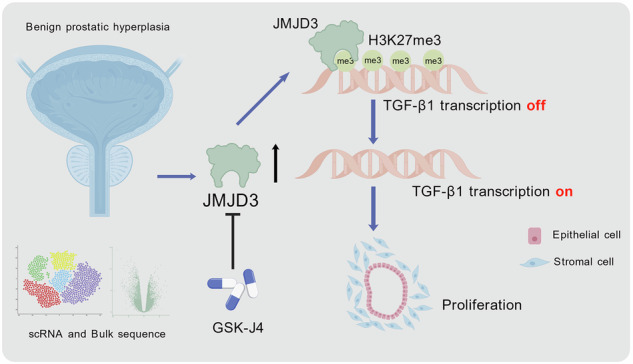
Our findings illustrated JMJD3’s contribution to BPH progression, and GSK-J4 offers a potential JMJD3-targeted therapy for BPH.

Our findings illustrated JMJD3’s contribution to BPH progression, and GSK-J4 offers a potential JMJD3-targeted therapy for BPH.

## Introduction

Benign prostatic hyperplasia (BPH) is a prevalent condition in aging male, characterized by bladder outlet obstruction-related symptoms and lower urinary tract symptoms (LUTS) such as hesitancy, weak urine stream, and incomplete emptying in urination [1]. The prevalence of BPH is markedly associated with age, presenting in 50–60% of male aged 60 and 80–90% of male aged 70 and older [2]. LUTS has a strong and negative impact on patient’s quality of life, even if features between individuals and therapy preferences are different as well [3]. The majority of patients with BPH present with the non-malignant enlargement of the prostate and an abnormal accumulation of epithelial and stromal cells in the periurethral area of the prostate [4]. In recent years, many efforts have been made to contribute to the pathogenesis of BPH, and the evidence demonstrated that inflammation storm of prostate [5], alterations of androgen and estrogen levels [6], interactions between stromal and epithelial cells et, al [7]. Nevertheless, the exact mechanisms of BPH development and progression remain unclear.

Currently, both 5α-reductase inhibitors and α-adrenergic receptor antagonists rank in the dominant place in the treatment of BPH. About 30% of the patients eventually will require surgical intervention due to disease progression [8]. Thus, to perform “the best treatment for the best patients” will promise the maximal benefit for patients with BPH. Then, it is necessary to illustrate the pathogenesis and exact molecular mechanisms of BPH.

Jumonji domain-containing protein D3 (JMJD3) belongs to the JmjC histone demethylase family and regulates gene expression by demethylating Histone 3 lysine 27 me3 (H3K27me3)[9, 10]. Under physiological conditions, JMJD3 expression remains at a low level in prostate tissues, whereas it can be strongly induced by various pathological stimuli, including inflammatory cytokines, oxidative stress, and carcinogenic factors [11, 12]. Various studies have revealed the critical roles of JMJD3 in human diseases, including immune diseases, developmental diseases, infectious diseases, and cancers [9]. Epigenetics refers to heritable regulation of gene expression without alterations in the DNA sequence itself [13]. As one of the major epigenetic regulation types, histone modifications, such as methylation, acetylation and ubiquitination, are reported to be essential for gene expression regulation and mediating various diseases through epigenetic control [14]. Histone 3 lysine 27 (H3K27) methylation represents a pivotal epigenetic mechanism, wherein its methylation or demethylation status actively regulates diverse biological processes such as cell renewal, disease initiation, and progression [4, 15, 16].

In the current study, we illustrated that JMJD3 was upregulated in BPH samples and was induced in vitro and in vivo by lipopolysaccharide (LPS). Further investigation showed that the functional roles of JMJD3 may be achieved by demethylating H3K27me3 at the promoter of TGF-β1. The in vivo BPH model confirmed the efficacy of GSK-J4 in suppressing disease progression. In summary, this study revealed an epigenetic regulatory mechanism in BPH and implied the potential of targeting JMJD3 for BPH treatment.

## Materials and methods

### Human samples

Normal prostate tissues were obtained from male patients (<40 years old) who underwent radical cystectomy, with pathological examination by two independent pathologists indicating no hyperplasia. Prostatic hyperplasia tissues were obtained from BPH patients receiving surgery, and were confirmed as hyperplasia prostate by postoperative pathological staining. This investigation was approved by the Ethics Review Committee of West China Hospital, and informed consent was obtained from all patients (No. 20230308039 and No. 2022-1700) in accordance with the Declaration of Helsinki.

### Animal models

All animal experiments were approved by the Experimental Animal Ethics Committee of West China Hospital of Sichuan University (Approval No. 20201211008) and conducted in accordance with institutional guidelines for animal welfare. Six-week-old male Sprague-Dawley rats were purchased from Chengdu Dossy Experimental Animals Co., Ltd. (Chengdu, China). After one-week acclimatization period, rats were randomly divided into three groups, including PBS group: PBS was injected into prostate (an equivalent volume of LPS) for once; LPS (Sigma, Cat#L4391) group: LPS (200 μg/kg) was injected into prostate for once; LPS + GSK-J4 group: LPS (200 μg/kg) was injected into prostate for once, and GSK-J4 (MCE, Cat# HY-15648B) was injected intraperitoneally at the dosage of 10 mg/kg every 2 days for 4 weeks (28 days).

Eight-week-old male C57BL/6 mice were purchased from Chengdu Dossy Experimental Animals Co., Ltd. (Chengdu, China). After one-week acclimatization period, they were randomly divided into three groups, including Control group: subcutaneously administered with corn oil, and intraperitoneally injected with 10% DMSO + 40% PEG300 + 5% Tween 80 + 45% Saline; BPH group: subcutaneously administered with testosterone propionate (TP) (Sigma, Cat#T1875) (dissolved in corn oil) 4 mg/kg/day; BPH + GSK-J4 group: subcutaneously administered with TP 4 mg/kg/day and intraperitoneally injected with GSK-J4 at the dosage of 10 mg/kg every 2 days for 4 weeks.

### Cell culture and cell transfection

BPH-1 cell was purchased from the Servicebio Co., Ltd (Wuhan, China) and cultured in RPMI-1640 medium (Thermo, USA) containing 10% FBS and 1% penicillin-streptomycin. WPMY-1 cell was purchased from the American Type Culture Collection (ATCC, Manassas, Virginia, USA), and cultured in DMEM medium (Thermo Fisher Scientific, MA, USA) containing 5% fetal bovine serum (FBS, Gibco, Australia) and 1% penicillin–streptomycin (HyClone). RWPE-1 cell were purchased from the American Type Culture Collection (ATCC, Manassas, Virginia, USA), and cultured in Keratinocyte Serum Free Medium (K-SFM) medium containing 0.05 mg/mL bovine pituitary extract (BPE) and 5 ng/mL human recombinant epidermal growth factor (EGF).

Cells were seeded in a 6-well plate and cultured overnight, and were transfected with pCMV-KDM6B(human)-3×HA-Neo plasmids (Miaoling Bio, Wuhan, China. Cat#P64311) or control vector for 72-96 h, using Lipo8000™ (Beyotime Bio, China. Cat#C0533). Then, cells were harvested for total RNA or protein extraction. JMJD3 knockdown was achieved by transfecting siRNA targeting *KDM6B* using Lipo8000™ (Beyotime Bio, China. Cat#C0533), and the sequences of siRNA were listed in Table S1.

### Cell counting kit-8 and Edu assay

Cell viability was assessed by Cell Counting Kit-8 (CCK8) assay. Cells (5 × 10^3^ cells per well) were plated into a 96-well plate and cultured overnight. Then, the medium in each well was replaced with 100 μL of fresh complete medium and treated with indicated interventions for 0, 24, 48, 72, or 96 h. Finally, adding CCK-8 solution (Oriscience Bio, Chengdu, China, Cat#CB101) (10 μL per well) to each well and incubating in the dark at 37 °C for 2 h, then the absorbance at 450 nm was measured using an Eon™ microplate reader (Bio-Tek, VT, USA).

Cell proliferation was detected by Edu assay. Cells (5 × 10^3^ cells per well) were plated into 96-well plate and cultured overnight. Then, the medium in each well was replaced with 100 μL of fresh complete medium and treated with the indicated interventions for 72 h. 5-Ethynyl-2-deoxyuridine (Edu) incorporation experiment was performed to detect cell proliferation according to the manufacture’s protocol (Elabscience, Wuhan, China. Cat#E-CK-A377). Briefly, cells were incubated with 50 μM Edu for 3 h before fixation, permeabilization, and Edu staining. The proportion of Edu-positive cells was determined by fluorescence microscope.

### Flow cytometry

Cells cultured in a 6-well plate were harvested into flow cytometry tubes. Cells were fixed in 500 μL of 75% ethanol at 4 °C overnight, washed, filtered, and then stained with PI/RNase dye working solution. After incubating at room temperature in the dark for 30 min. Then, cell cycle was detected by using the cell cycle kit (KeyGen, Jiangsu, China, Cat#KGA9101) according to the manufacturer’s instructions. Cell apoptosis was directly determined by using the Annexin V-APC/PI apoptosis kit (Elabscience, Wuhan, China, Cat#E-K-A217). Cells were resuspended in 500 μL of 1× binding buffer, incubated with 5 μL of Annexin V-APC and 5 μL of PI Reagent at room temperature in the dark for 15-20 min, and then apoptosis was evaluated by using the flow cytometer.

Cytoplasmic Reactive Oxygen Species (ROS) was detected by using ROS Fluorometric Assay Kit (Elabscience, Wuhan, China. Cat#E-BC-F005) according to the manufacturer’s instructions.

### Real-time quantitative polymerase chain reaction (RT-qPCR)

Total RNA was extracted by using Total RNA Isolation Kit (FOREGENE, Chengdu, China, Cat#RE-03113). cDNA was prepared according to the protocol and subjected to real-time quantitative PCR (RT-PCR) using SYBER Green mix according to the manufacturer’s instructions. Each reaction was normalized to the co-amplification of GAPDH. The primer sequences for RT-qPCR were listed in Table S2.

### Western blotting

Whole cell protein extracts were resolved on SDS-PAGE, and proteins were transferred to PVDF membranes. 5% milk in PBS/0.1% Tween-20 was used for blocking for 1 h at room temperature. Then, membranes were incubated overnight at 4 °C with the indicated primary antibodies and secondary antibodies (Anti-Rabbit or Anti-Mouse). The antigen-antibody reaction was detected using an enhanced chemiluminescence (ECL) detection kit (Millipore, MA, USA. Cat#WBKLS0500). The details of primary antibodies for western blotting were listed in Table S3.

### Cell immunofluorescence (IF)

Cells were seeded in a 96-well plate and cultured overnight. Then, cells were fixed with 4% paraformaldehyde for 30 min, permeabilized with 0.1% Triton X-100 for 5 min, and incubated with 5% goat serum at 37 °C for 30 min. Cells were incubated with anti-JMJD3 antibody (Table S3) overnight. Intracellular JMJD3 was visualized by using FITC-conjugated secondary antibodies, and nuclei were visualized with DAPI.

### Histological staining

Tissues were fixed in 4% paraformaldehyde and embedded in paraffin. The tissues were then sectioned into 4-μm-thick slices. Subsequently, we stained the sections with hematoxylin and eosin (HE) to evaluate histopathological features. Inflammatory scoring of prostate tissue via HE staining was performed without knowledge of the patients’ clinical information. The scoring was based on a combination of the inflammatory infiltration area (scored 1–4 for 0–25%, 26–50%, 51–75%, and 76–100%, respectively) and the intensity of inflammatory infiltration (scored 1 for no infiltration, 2 for mild infiltration, 3 for moderate infiltration, and 4 for severe infiltration, respectively).

### Immunohistochemistry (IHC)

Prostate tissue samples were deparaffinized and hydrated, followed by overnight incubation with primary antibodies at 4 °C. After incubation with corresponding secondary antibodies, sections were stained with diaminobenzidine (DAB) and counterstained with hematoxylin. Quantitative analyses were performed without awareness of patient clinical information by combining the area percent of positive cells from 1 to 4 (1 is 0–25%, 2 is 26–50%, 3 is 51–75%, and 4 is 76–100%) and the relative intensity of the staining gray level from 1 to 4 (1 is negative, 2 is low, 3 is medium, and 4 is high). All statistical significance was obtained through the z-score calculator for 2 population proportions (https://www.socscistatistics.com/tests/ztest/) between a group with scores 1 and 2(negative to low intensity) and a group with scores 3 and 4 (medium to high intensity). The detailed information of primary antibodies for IHC staining were listed in Table S3.

### Bulk RNA sequencing analysis

The total RNA of vector and JMJD3-overexpression RWPE-1 and WPMY-1 cells was extracted by Total RNA Isolation Kit (FOREGENE, Chengdu, China) and underwent DNase digestion following the manufacturer’s instructions. RNA-seq libraries from 1 μg of total RNA were prepared using the Illuminia Tru-Seq RNA Sample. The mRNA-Seq paired-end library was prepared using Illumina NGS on a HiSeq 4000:2 ×150 cycles/bases (150 bp, PE). Around 30 M of reads/sample were obtained. Data analysis was performed using a Top Hat-Cufflinks pipeline, and sequence read mapping/alignment was performed using HISAT. StringTie Data were mapped and quantified for 27,044 unique genes/transcripts. Gene and transcript expression was quantified as FPKM (Fragments Per Kilobase of transcript per million mapped reads).

### GEO datasets

The Bulk RNA-seq, Single cell RNA-seq and CHIP-seq raw data are available at Gene Expression Omnibus (GEO) with the accession numbers GSE168777, GSE255550, GSE138157 and GSE172357, respectively.

### scRNA- sequencing dataset analysis

For the single-cell RNA sequencing dataset GSE172357 (comprising 13 benign prostatic hyperplasia and 6 normal prostate tissue samples), Seurat software (version 5.4.0) was employed for data processing. Quality control was applied to retain cells expressing 250–5000 genes, with mitochondrial gene content below 10% and hemoglobin gene expression under 1%. Doublets were computationally identified and removed using DoubletFinder. Normalization was conducted with SCTransform, followed by dimensionality reduction via principal component analysis (top 40 principal components). Clustering was performed at a resolution of 0.7, and the results were visualized using Uniform Manifold Approximation and Projection. Cluster-specific marker genes were identified with FindAllMarkers, and cell type annotation was carried out based on established canonical markers.

### Statistical analysis

The data are presented as means ± standard deviation of the mean (SD) from three independent experiments. Statistical analyses were performed with GraphPad Prism 9.0. Differences between individual groups were analyzed by two tailed Student’s *t* test or one-way analysis of variance (ANOVA) followed by the Scheffé procedure for comparison of means. *p* < 0.05 was considered statistically significant.

## Results

### JMJD3 is upregulated in benign prostatic hyperplasia

**Fig. 1:**
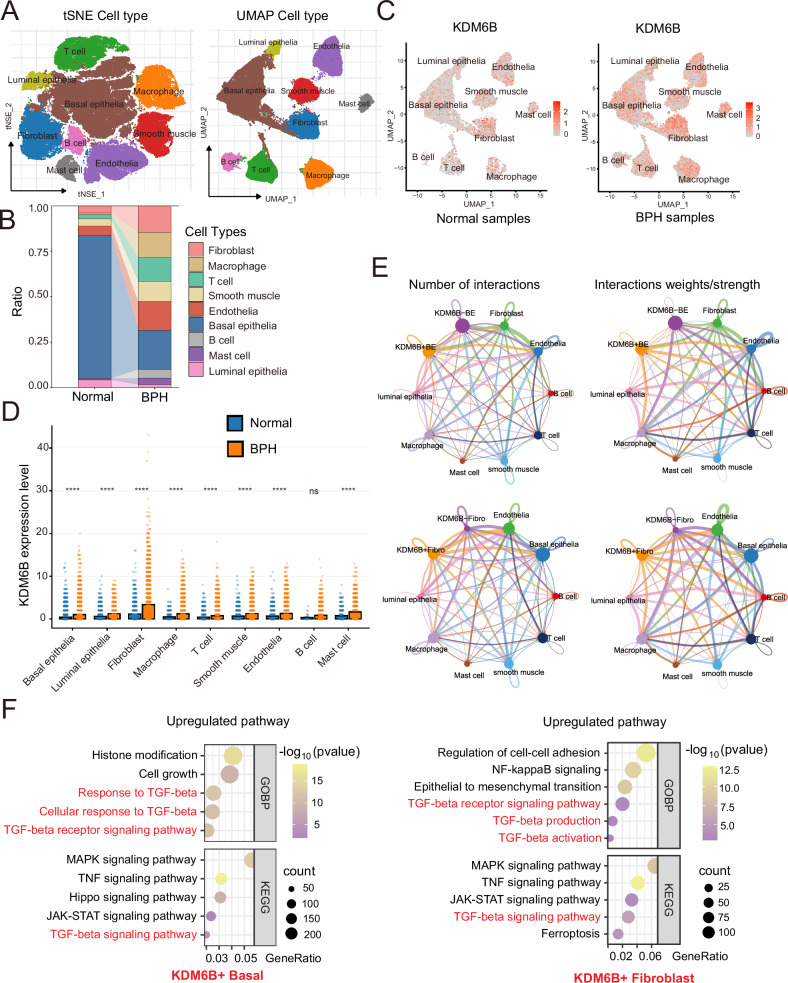
Single-cell RNA sequencing reveals upregulation of KDM6B (JMJD3) in benign prostatic hyperplasia. **A** tNSE and UMAP plots showed 9 cell types derived from single-cell sequence data in normal and BPH samples. **B** Proportion distribution of each cell type between normal and BPH prostate tissues. **C** UMAP plots showed the distribution of KDM6B expression among different cell types in normal and BPH prostate tissues. **D** Bar plot showing the expression levels of KDM6B among different cell types in normal and BPH prostate tissues. **E** Inferred intercellular communication networks using CellChat. The left panel shows the number of ligand-receptor interactions between cell types, while the right panel indicates the interaction strength/weights. **F** Pathway enrichment analysis of upregulated genes in KDM6B+ basal cells and KDM6B+ fibroblasts from BPH samples.

To investigate the expression of *KDM6B* (JMJD3) in normal and BPH prostate tissues from human samples, we downloaded the single-cell RNA sequence data from the GEO database via the accession number GSE172357. Post-processing, we identified 38874 cells in normal prostate tissues and 85742 cells in BPH prostate tissues, including 9 cell types (Fig. 1A), which were classified into 23 cell clusters (Fig. S1A). The relative composition of recovered cell populations, such as fibroblast, macrophage, T cell, smooth muscle cell, endothelial cell, B cell and mast cell, differed between the normal and BPH group. (Fig. 1B). However, because cell proportions in scRNA-seq datasets can be influenced by tissue sampling, dissociation efficiency, cell survival, capture efficiency, and quality-control filtering, these distributions should be interpreted as the composition of recovered cells in this dataset rather than direct estimates of histological abundance. Further analysis indicated that the mRNA expression levels of *KDM6B* were significantly upregulated among fibroblast, basal and luminal epithelial cells in the BPH group compared to the normal group, respectively (Fig. 1C, D). Then, inferred intercellular communication networks using CellChat analysis, strong signaling crosstalk was observed among immune cells, stromal cells, and epithelial populations (Figs. 1E and S1B) in BPH prostate samples. In addition, all the cells were divided into KDM6B+ and KDM6B- cells according to the mRNA expression levels of *KDM6B* in BPH prostate tissues. Volcano plots displayed differentially expressed genes (|Log2Foldchange | ≥0.5; *P* < 0.05) between KDM6B+ and KDM6B- cells across all BPH cells, basal cells, luminal cells, and fibroblast cells from BPH samples (Fig. S1C). Pathway enrichment analysis revealed distinct signaling patterns across KDM6B+ cell populations in BPH samples. Upregulated pathways included histone modification, cell growth, response to TGF-β, cellular response to TGF-β, TGF-β receptor signaling pathway and others in KDM6B+ basal epithelial cells from BPH samples, as well as regulation of cell-cell adhesion, NF-κB signaling, TGF- β signaling pathway, JAK-STAT signaling pathway and others in KDM6B+ fibroblast cells from BPH samples, as well as histone modification, Wnt signaling, TGF-β signaling, ERBB signaling, Hippo signaling, and others in KDM6B+ luminal cells from BPH samples (Figs. 1F and S1D).

**Fig. 2:**
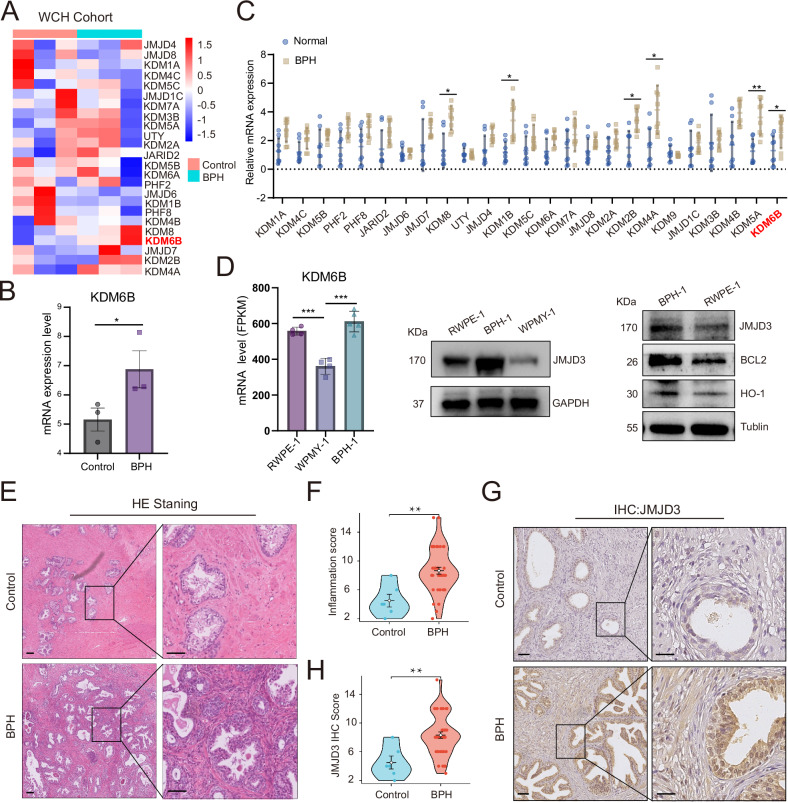
Bulk RNA sequencing and clinical samples reveal upregulation of JMJD3 in benign prostatic hyperplasia. **A, B** The total RNA of prostates was harvested from control (*n* = 3) and benign prostatic hyperplasia (BPH) (*n* = 3) patients for Bulk RNA sequencing. Heatmap was used to show the mRNA levels of histone demethylases in control and BPH samples, and bar plot showing the mRNA expression of *KDM6B* in control and BPH samples. **C** RT-PCR was used to test the mRNA levels of histone demethylases in control (*n* = 8) and BPH (*n* = 8) samples. **D** Bulk sequence was performed to test the mRNA expressions of *KDM6B* in RWPE-1, WPMY-1, and BPH-1 cells. Western blot was performed to test the protein levels of JMJD3 in RWPE-1, WPMY-1, and BPH-1 cells. **E, F** Hematoxylin-eosin (HE) staining was used to determine the pathological features of prostates in control (*n* = 6) and benign prostatic hyperplasia (*n* = 41) patients. **G, H** Immunohistochemistry (IHC) staining was performed to test the protein levels of JMJD3 in control (*n* = 6) and benign prostatic hyperplasia (*n* = 41) patients.

Total RNA for Bulk RNA-seq was extracted from control and BPH prostate tissues obtained from the West China Hospital of Sichuan University. Heatmap was used to show the mRNA expressions of a total of 26 histone demethylases, including *JMJD4*, *JMJD8*, and *KDM6B* in control (*n* = 3) and BPH (*n* = 3) (Fig. 2A). The mRNA levels of *KDM6B* were significantly upregulated in BPH compared with control (*P* < 0.05) (Fig. 2B). Then, the results of RT-PCR showed that the mRNA levels of *KDM8*, *KDM1B*, *KDM2B*, *KDM4A*, *KDM5A*, and *KDM6B* were significantly upregulated in BPH (*n* = 8) compared with control (*n* = 8) (all *P* < 0.05) (Fig. 2C). The results of Bulk RNA sequence revealed that both RWPE-1 and BPH-1 cells show higher *KDM6B* mRNA levels comparing to WPMY-1 cell, respectively (all *P* < 0.001) (Fig. 2D). Then, JMJD3 protein levels were markedly elevated in BPH-1 cells compared with RWPE-1 and WPMY-1 cells, and the anti-apoptotic protein BCL2 and the oxidative stress-related protein HO-1 were also upregulated in BPH-1 cells compared to RWPE-1 cells (Fig. 2D). Immunofluorescence staining revealed the presence of JMJD3 in both WPMY-1 and BPH-1 cells, respectively (Fig. S2A, B). Human normal (*n* = 8) and BPH (*n* = 41) prostate tissues were harvested from West China Hospital. Hematoxylin-eosin (HE) staining illustrated higher levels of inflammation in BPH prostate tissues compared with normal prostate tissues (*P* < 0.01) (Fig. 2E, F). In addition, immunohistochemistry (IHC) staining revealed a significant upregulation of JMJD3 in the BPH group compared to the normal group (*P* <0.01) (Fig. 2G, H). Taken together, these results suggested that JMJD3 is significantly upregulated in BPH and BPH prostate tissues show higher levels of inflammation.

### LPS induces an inflammation response and upregulates JMJD3 in vitro and in vivo

**Fig. 3:**
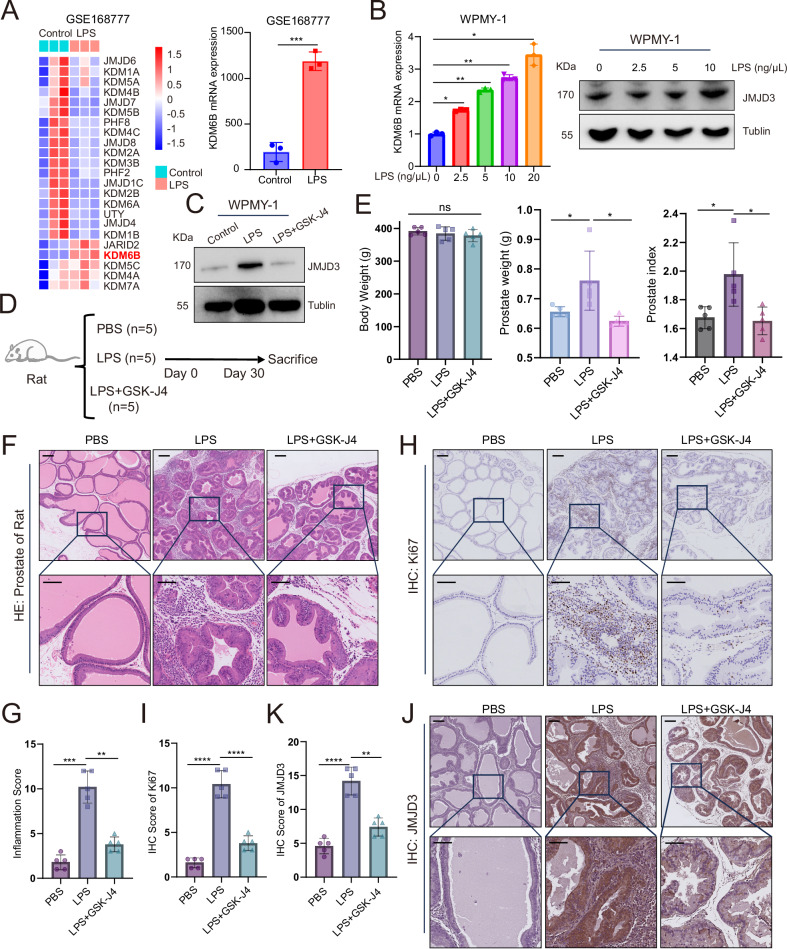
LPS induces inflammatory responses and upregulates JMJD3 expression in vitro and in vivo. **A** Heatmap was used to show the mRNA expressions of histone demethylases in control and LPS group from GSE168777 dataset. **B** WPMY-1 cells were treated with doses of LPS (0, 2.5, 5, 10, or 20 ng/μL) for 72 h. Then, RT-PCR and western blot were performed to determine the mRNA and protein levels of JMJD3 in WPMY-1 cells, respectively. **C** WPMY-1 cells were treated with DMSO, LPS (10 ng/μL), or LPS (10 ng/μL) plus GSK-J4 (7.5 μM). Then, Western blot was used to test the protein levels of JMJD3. **D** Flowchart shows the experimental procedures of rats receiving LPS and GSK-J4 treatment. Six-week-old male Sprague-Dawley rats were randomly divided into 3 groups, including PBS group (*n* = 5), LPS group (*n* = 5), and LPS + GSK-J4 group (*n* = 5). **E** Bar plots showing the body weight, prostate weight, and prostate index in PBS, LPS, and LPS + GSK-J4 group, respectively. **F, G** Representative HE staining and quantification in PBS, LPS, and LPS + GSK-J4 group, respectively. **H**–**K** Representative IHC Ki67 and JMJD3 staining and quantification in PBS, LPS, and LPS + GSK-J4 group, respectively.

The Bulk RNA-seq dataset GSE168777 was downloaded from the Gene Expression Omnibus (GEO) and analyzed. The resulting heatmap clearly displayed the expression profiles of 26 histone demethylases in control versus LPS-treated conditions (Fig. 3A). And the bar plot showed that the mRNA levels of *KDM6B* are significantly upregulated by LPS treatment compared with control group (*P* <0.001) (Fig. 3A). Both RT-PCR and Western blot analyses showed that LPS treatment upregulated JMJD3 expression in a dose-dependent manner in WPMY-1 cells. Specifically, JMJD3 mRNA and protein levels increased significantly with higher LPS concentrations compared to the untreated control (all *P* < 0.05) (Fig. 3B). Western blot analysis demonstrated that pretreatment with the JMJD3 inhibitor GSK-J4 (7.5 μM) partially attenuated the LPS-induced upregulation of JMJD3 protein expression (Fig. 3C).

To complement our in vitro findings, we conducted in vivo experiments to validate the functional role of LPS-induced JMJD3 expression in the pathogenesis of BPH (Fig. 3D). Prostate enlargement was induced in the in vivo model by administering LPS to rats over a four-week period. This LPS-BPH model resulted in a significant increase in both absolute prostate weight and the prostate index, whereas no significant change was observed in the overall body weight of the rats (Fig. 3E). Histological assessment through HE staining revealed that prostate tissues from LPS-BPH rats exhibited significant morphological alterations compared to the PBS-treated control group. Notably, there was a marked expansion in both the epithelial and stromal compartments, characterized by increased cellularity and overall glandular hypertrophy (Fig. 3F, G). Furthermore, IHC staining demonstrated elevated expression of the proliferation marker Ki67 in prostate tissues from LPS-treated rats. The increased Ki67 signal was predominantly localized within the stromal compartment. This LPS-induced proliferative response was significantly attenuated by concurrent administration of JMJD3 inhibitor, GSK-J4 (Fig. 3H, I). Subsequent IHC staining revealed that a significant increase in JMJD3 protein expression within the prostate tissues of LPS-treated rats, predominantly in the epithelium, partially in the stromal, which was effectively suppressed by GSK-J4 (Fig. 3J, K).

### JMJD3 overexpression promotes cell proliferation and accelerates cell cycle progression in RWPE-1 and WPMY-1 cells

**Fig. 4:**
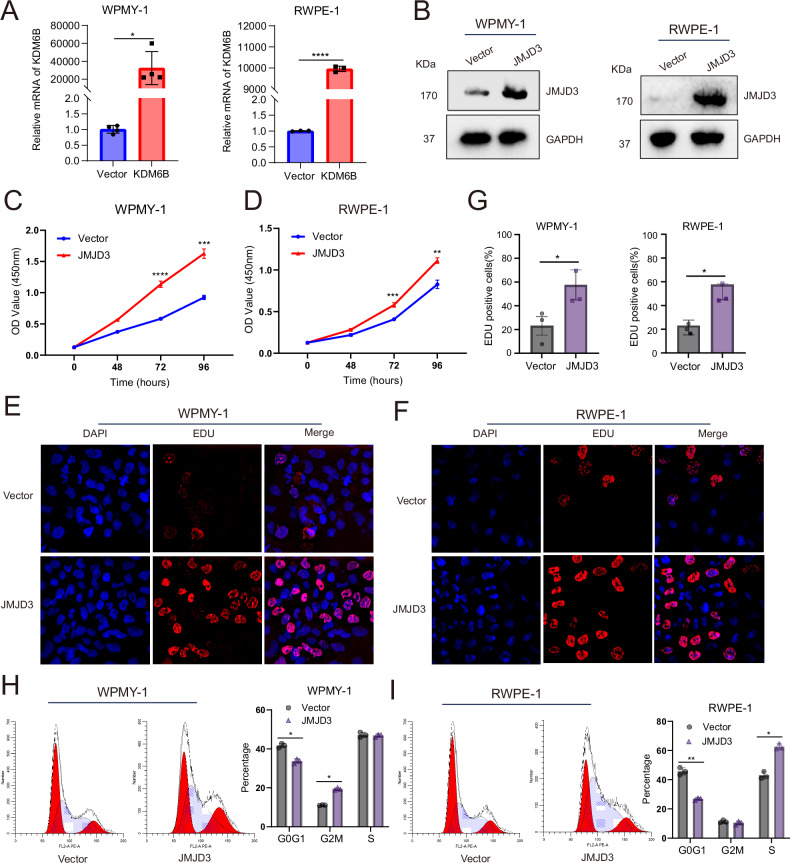
JMJD3 overexpression enhances cell viability and modulates the cell cycle in prostate cells. **A**, **B** RT-PCR and Western blot were performed to confirm the JMJD3 overexpression effects in WPMY-1 and RWPE-1 cells after transfected with vector or *KDM6B* plasmids for 3 days, respectively. **C, D** WPMY-1 (3000 cells/well) and RWPE-1 (5000 cells/well) cells were plated in 96-well plates and transfected with vector or *KDM6B* plasmids for 0, 48, 72, or 96 h, respectively. Cells viability was determined by CCK-8 assay. **E**–**G**. WPMY-1 (3000 cells/well) and RWPE-1 (5000 cells/well) cells were plated in 96-well plates and transfected with vector or *KDM6B* plasmids for 72 h, respectively. Then, cells proliferation was tested by EdU assay. **H, I**. WPMY-1 and RWPE-1 cells were plated in 60 mm dishes, and transfected with vector or *KDM6B* overexpression plasmids for 3 days, respectively. Then, cell cycles were determined by flow cytometry, respectively.

To overexpress JMJD3 in prostate cells, we transfected *KDM6B* expression plasmids into WPMY-1 and RWPE-1 cells. lines RT-PCR and Western blot analyses confirmed that JMJD3 was successfully overexpressed at both the mRNA and protein levels in WPMY-1 and RWPE-1 cells compared to their respective controls (all *P* < 0.05), respectively (Fig. 4A, B). CCK-8 assays revealed that transfection with *KDM6B* expression plasmids significantly enhanced the proliferative capacity of both WPMY-1 and RWPE-1 cells at 72 and 96 h post-transfection compared to the control groups (all *P* < 0.05) (Fig. 4C, D). Furthermore, the results of EDU assays indicated that overexpressing *KDM6B* promotes the proliferation of both WPMY-1 and RWPE-1 cells (all *P* < 0.05) (Fig. 4E–G). In addition, the flow cytometry analysis revealed that overexpression of *KDM6B* could significantly reduce the percentage of cells in G0/G1 phase while concurrently increasing the percentage in G2/M phase compared to vector group in WPMY-1 cells (all *P* < 0.05) (Fig. 4H). Similarly, in RWPE-1 cells, *KDM6B* overexpression significantly promoted cell cycle progression, evidenced by a reduced proportion of cells in G0/G1 phase and an increased proportion in S phase (all *P* < 0.05) (Fig. 4I).

### The JMJD3 inhibition attenuates proliferation, induces cell cycle arrest, and promotes apoptosis in BPH-1 cells

**Fig. 5:**
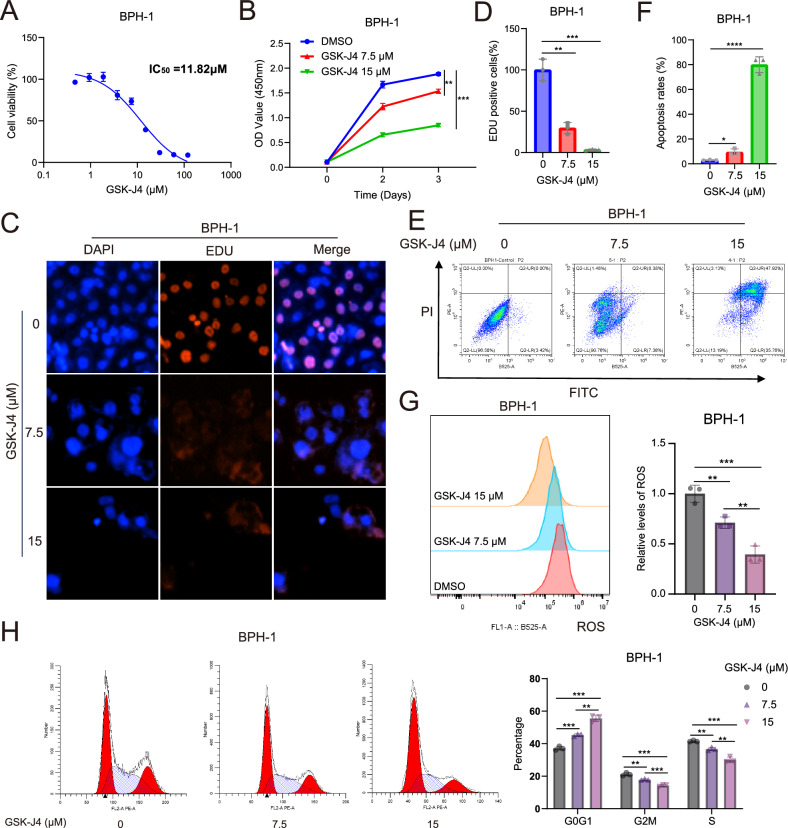
Pharmacological inhibition of JMJD3 reduces cell viability and induces cell cycle arrest and apoptosis in prostate cells. **A** BPH-1 (3000 cells/well) cells were plated in 96-well plate and cultured overnight. Then, BPH-1 cells were treated with doses of JMJD3 inhibitor GSK-J4 (0, 0.47, 0.94, 1.88, 3.75, 7.5, 15, 30, 60, or 120 μM) for 48 h, and cell viability was determined by CCK-8 assay. **B** BPH-1 (3000 cells/well) cells were plated in 96-well plate and cultured overnight. Then, BPH-1 cells were treated with doses of JMJD3 inhibitor GSK-J4 (0, 7.5, or 15 μM) for 0, 48, or 72 h, and cell viability was determined by CCK-8 assay, respectively. **C, D** BPH-1 (3000 cells/well) cells were plated in a 96-well plate and cultured overnight. Then, BPH-1 cells were treated with doses of JMJD3 inhibitor GSK-J4 (0, 7.5, or 15 μM) for 48 h, and cell proliferation was tested by EdU assay, respectively. **E, F** BPH-1 (50 × 10^4^) cells were plated in 60 cm dishes and cultured overnight. Then, BPH-1 cells were treated with DMSO, or GSK-J4 (7.5, or 15 μM) for 48 h. After GSK-J4 treatment, cells were harvested and apoptosis of cells were determined by flowcytometry, respectively. **G, H** BPH-1 (50*10^4) cells were plated in 60 cm dishes and cultured overnight. Then, BPH-1 cells were treated with DMSO, or GSK-J4 (7.5, or 15 μM) for 48 h. After GSK-J4 treatment, cytoplasmic ROS of cells and cell cycles were determined by flow cytometry, respectively.

To investigate the functional impact of JMJD3 blocking, we employed its inhibitor, GSK-J4, to assess its effects on key cellular processes in BPH-1 cells, including proliferation, cell cycle progression, apoptosis, and cytoplasmic reactive oxygen species (ROS) generation. Initial cell viability assays determined the inhibitory effect of GSK-J4 on BPH-1 cell proliferation, yielding a half-maximal inhibitory concentration (IC_50_) of 11.82 μM (Fig. 5A). CCK-8 analysis demonstrated that GSK-J4 treatment inhibited the viability of BPH-1 cells in dose-dependent and time-dependent manners (all *P* < 0.05) (Fig. 5B). This anti-proliferative effect was further validated by EdU incorporation assays, which demonstrated a significant reduction in the proportion of proliferating BPH-1 cells following GSK-J4 treatment (Fig. 5C, D). Additionally, GSK-J4 promoted apoptosis of BPH-1 cells in a dose-dependent manner (all *P* < 0.05) (Fig. 5E, F). Furthermore, GSK-J4 treatment led to a dose-dependent reduction in the levels of cytoplasmic reactive oxygen species (ROS) within BPH-1 cells (all *p* < 0.01) (Fig. 5G). To determine the effect of JMJD3 inhibition on cell cycle distribution, flow cytometry analysis was performed on BPH-1 cells. The results indicated that GSK-J4 treatment induced a dose-dependent accumulation of cells in the G0/G1 phase, concomitant with a corresponding decrease in the proportion of cells in both the S and G2/M phases (all *p* < 0.01) (Fig. 5H).

Furthermore, we performed siRNA-mediated knockdown in BPH-1 cells. RT–PCR analysis demonstrated that two independent siRNAs (siKDM6B#1 and siKDM6B#3) efficiently reduced KDM6B mRNA expression compared with siControl (all *P* < 0.01) (Fig. S3A). Consistently, western blot analysis confirmed a marked decrease in JMJD3 protein levels following KDM6B silencing (Fig. S3B). Functional assays revealed that *KDM6B* knockdown significantly impaired cell proliferation. CCK-8 assays showed a time-dependent reduction in cell viability, with significant suppression observed at 48 h post-transfection (*P* < 0.01) (Fig. S3C). EdU incorporation assays further demonstrated a marked decrease in DNA synthesis in *KDM6B*-silenced cells, as evidenced by reduced EdU-positive nuclei (all *P* < 0.01) (Fig. S3D, E), indicating diminished proliferative activity. Then, *KDM6B* knockdown led to a significant accumulation of cells in the G0/G1 phase, accompanied by a reduction in G2M-phase populations (*P* < 0.05) (Fig. S3F). Moreover, Annexin V/PI staining demonstrated that silencing *KDM6B* significantly increased apoptotic cell populations compared with control cells (*P* < 0.01) (Fig. S3G).

### JMJD3 overexpression induces gene expression reprogramming in RWPE-1 and WPMY-1 cells

**Fig. 6:**
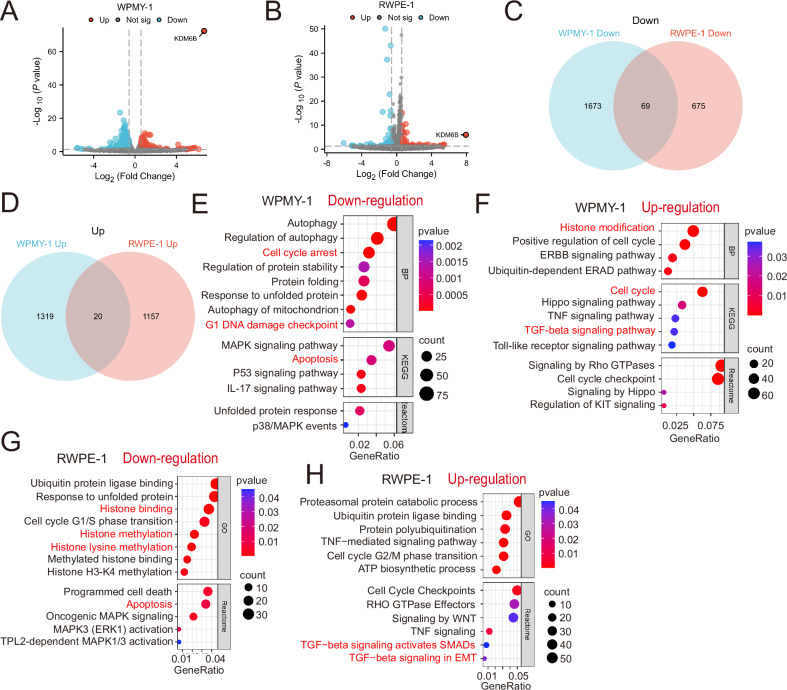
JMJD3 overexpression drives transcriptional reprogramming in prostate cells. **A, B** WPMY-1 and RWPE-1 cells were plated in 60 mm dishes, and transfected with vector or *KDM6B* expression plasmids for 3 days, respectively. Then, Total RNAs were extracted for Bulk RNA sequencing. Valcano plots showing the differentially expressed genes in WPMY-1 cells and RWPE-1 cells, respectively. Blue dots represent down-regulated, and red dots represent up-regulated (2-fold and *P* < 0.05) genes. **C, D** Venn plots showing co-downregulated and co-upregulated genes in WPMY-1 cells and RWPE-1 cells, respectively. **E, F** Gene Ontology (GO), Kyoto Encyclopedia of Genes and Genomes (KEGG), and Reactome analyses illustrate the up-regulated and down-regulated pathways in WPMY-1 cells transfected with vector or *KDM6B* expression plasmids for 72 h, respectively. **G, H** Gene Ontology (GO), Kyoto Encyclopedia of Genes and Genomes (KEGG), and Reactome analyses illustrate the up-regulated and down-regulated pathways in RWPE-1 cells transfected with vector or *KDM6B* expression plasmids for 72 h, respectively.

To investigate the transcriptional impact of *KDM6B* overexpression in prostate cells, both WPMY-1 and RWPE-1 cells were transfected with *KDM6B* expression plasmids for 72 h. Then, the total RNA was extracted for Bulk RNA sequencing. Valcano plot revealed that a total of 1742 genes are downregulated and a total of 1339 genes are upregulated in WPMY-1 cells (|Foldchange | ≥2, *P* <0.05) (Fig. 6A). And a total of 744 genes were downregulated and a total of 1177 genes were upregulated in RWPE-1 cells (|Foldchange | ≥2, *P* < 0.05) (Fig. 6B). Further analysis indicated that 69 genes are co-downregulated and 20 genes are co-upregulated in WPMY-1 and RWPE-1 cells, respectively (Fig. 6C, D). Subsequent analysis revealed that 14 pathways are downregulated in WPMY-1 cells with *KDM6B* overexpression compared to the vector, including autophagy, regulation of autophagy, cell cycle arrest, regulation of protein stability, protein folding, response to unfolded protein, autophagy of mitochondrion, G1 DNA damage checkpoint, MAPK signaling pathway, apoptosis, P53 signal pathway, IL-17 signal pathway, et, al. (all *P* < 0.05) (Fig. 6E). Then, 13 pathways are upregulated in WPMY-1 cells with KDM6B overexpression compared to vector, including histone modification, cell cycle, TGF-beta pathway, et, al. (all *P* < 0.05) (Fig. 6F). Further analysis demonstrated that 13 pathways are downregulated in RWPE-1 cells with *KDM6B* overexpression compared to vector, including histone binding, histone methylation, histone lysine methylation, apoptosis, et, al. (all *P* < 0.05) (Fig. 6G). In addition, 12 pathways are upregulated in RWPE-1 cells with *KDM6B* overexpression compared to vector, including TGF-beta signaling activates SMADs, TGF-beta signaling in EMT, cell cycle G2/M phase transition, et, al. (all *P* < 0.05) (Fig. 6H). GSEA analysis demonstrated that demethylase activity and HMDS demethylate histones are upregulated, and G1 phase is downregulated in WPMY-1 cells transfected with *KDM6B* expression plasmids (all *P* < 0.05) (Fig. S4A). GSEA analysis also revealed that demethylation, demethylase activity, and methyltransferase complex are upregulated in RWPE-1 cells with *KDM6B* overexpression (all *P* < 0.05) (Fig. S4B).

### JMJD3 activates TGF-β signaling through H3K27me3 demethylation

**Fig. 7:**
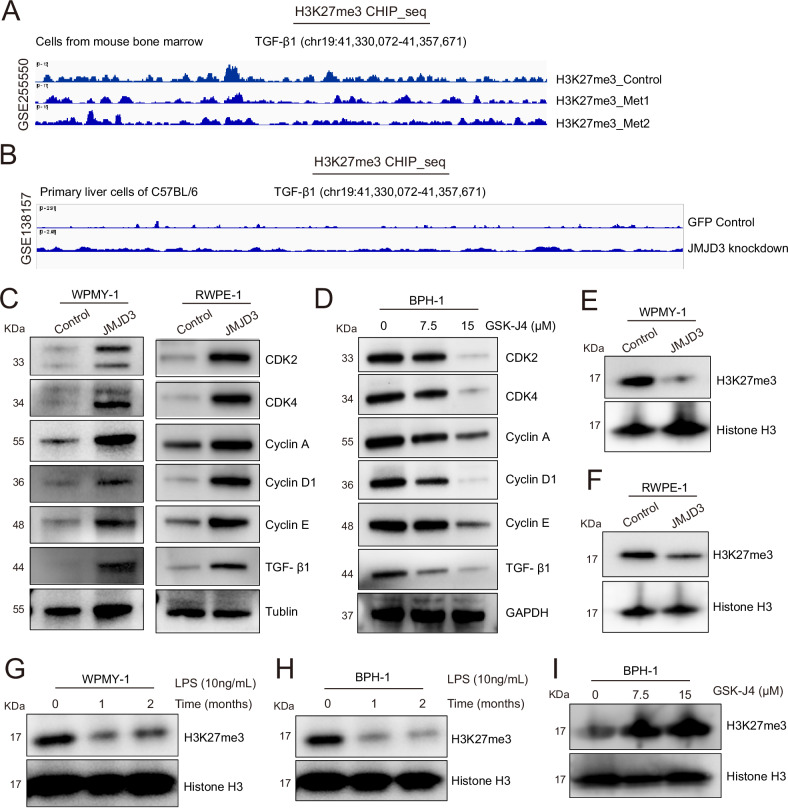
JMJD3 activates TGF-β1 signaling in a H3K27me3-dependent manner. **A** CHIP sequencing of histone H3K27 trimethylation (H3K27me3) in GSE255550 dataset was performed with mouse bone marrow cells. And the plot showing the normalized H3K27-me3 peaks at *TGF-β1*. **B** CHIP sequencing of H3K27me3 in GSE138157 dataset was performed with primary liver cells of C57BL/6 mouse. And the plot showing the normalized H3K27-me3 peaks at *TGF-β1*. **C** WPMY-1 and RWPE-1 cells were plated in 60 mm dishes, and transfected with vector or *KDM6B* expression plasmids for 3 days, respectively. Then, whole cell lysates were harvested for determining the protein levels of CDK2, CDK4, Cyclin A, Cyclin D1, Cyclin E and TGF-β1. Tublin was used as internal control, respectively. **D** BPH-1 cells were plated in 60 mm dishes and cultured overnight. Then, BPH-1 cells were treated with DMSO or JMJD3 inhibitor GSK-J4 (7.5, 15 μM) for 3 days. Western blot was performed to test the protein levels of CDK2, CDK4, Cyclin A, Cyclin D1, Cyclin E and TGF-β1. GAPDH was used as internal control, respectively. **E, F** Western blot was performed to determine the protein levels of H3K27me3 in WPMY-1 and RWPE-1 cells after overexpressing JMJD3, respectively. Histone H3 was used as internal control. **G, H** WPMY-1 and BPH-1 cells were treated with 10 ng/mL Lipopolysaccharides (LPS) for 0, 1, or 2 months, respectively. Then, whole cell lysates were harvested to determine the protein levels of H3K27me3. Histone H3 was used as an internal control. **I** BPH-1 cells were plated in 60 mm dishes and cultured overnight. Then, BPH-1 cells were treated with DMSO or JMJD3 inhibitor GSK-J4 (7.5, 15 μM) for 3 days. A Western blot was performed to test the protein levels of H3K27me3. Histone H3 was used as an internal control.

Bulk RNA sequencing analysis revealed a transcriptional profile characterized by downregulated histone methylation pathways and upregulated TGF-β signaling in both WPMY-1 and RWPE-1 cells following *KDM6B* overexpression. Interrogation of publicly available ChIP-seq data (GSE255550) demonstrated that the *TGF-β1* and *TGF-β3* gene loci, but not *TGF-β2*, were marked by H3K27me3 (Figs. 7A and S5A). Complementary data from an independent dataset (GSE138157) indicated that JMJD3 knockdown led to an increase in H3K27me3 enrichment specifically at the *TGF-β1* locus, with no significant change observed at the *TGF-β3* locus (Figs. 7B and S5B). Consistent with a role in cell cycle progression, JMJD3 overexpression resulted in elevated protein expression of key regulators, including CDK2, CDK4, Cyclin A, Cyclin D1, Cyclin E, and TGF-β1, in both WPMY-1 and RWPE-1 cells (Fig. 7C). Conversely, inhibition of JMJD3 with GSK-J4 significantly reduced the expression of these same proteins in BPH-1 cells (Fig. 7D). Direct assessment of the histone mark confirmed the demethylase activity of JMJD3. Global levels of H3K27me3 were decreased in both WPMY-1 and RWPE-1 cells upon JMJD3 overexpression compared to vector controls (Fig. 7E, F). This reduction in H3K27me3 was also recapitulated by lipopolysaccharide (LPS) treatment in WPMY-1 and BPH-1 cells (Fig. 7G, H), linking an inflammatory stimulus to JMJD3-mediated epigenetic remodeling. Finally, treatment with the JMJD3 inhibitor GSK-J4 effectively increased global H3K27me3 levels in BPH-1 cells (Fig. 7I). Collectively, these data establish a mechanism whereby JMJD3 promotes TGF-β signaling and cell cycle gene expression by catalyzing the demethylation of the repressive H3K27me3 mark.

### JMJD3 inhibitor GSK-J4 exhibits therapeutic efficacy in a benign prostatic hyperplasia in vivo model

**Fig. 8:**
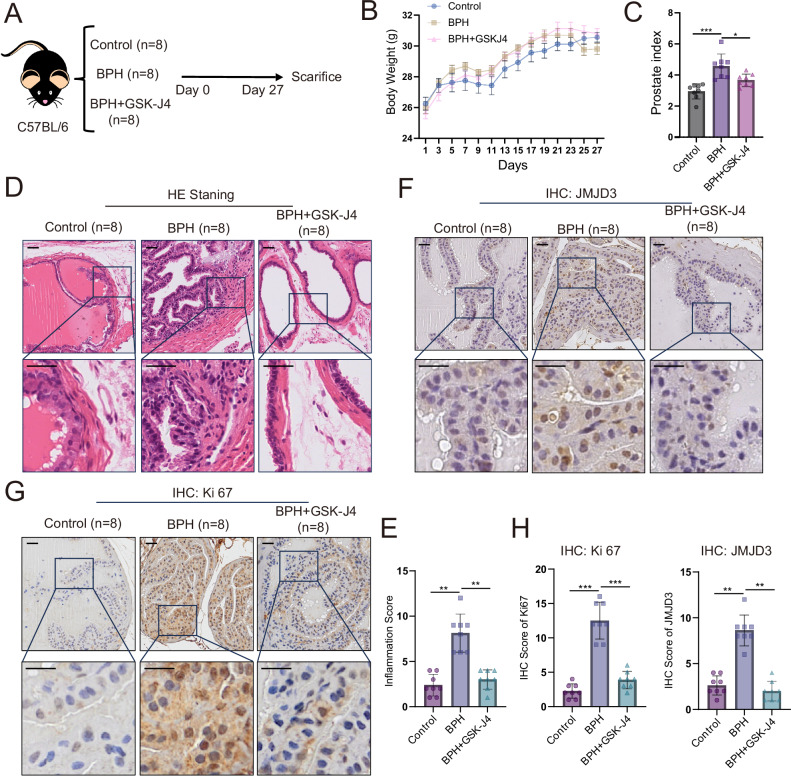
JMJD3 inhibitor GSK-J4 attenuates benign prostatic hyperplasia in vivo. **A** Experimental outline illustrating that C57BL/6 mice were randomly divided into 3 groups, including Control group (*n* = 8), BPH group (*n* = 8), and BPH + GSK-J4 group (*n* = 8). **B** Growth curves show the body weight of C57BL/6 mice. **C** Bar plot shows prostate index in Control, BPH, and BPH + GSK-J4 groups. **D, E** Representative HE staining in Control, BPH, and BPH + GSK-J4 group, respectively. **F, G** Representative IHC JMJD3 and Ki67 staining Control, BPH, and BPH + GSK-J4 group, respectively. **H** Quantifications for IHC scores of JMJD3 and Ki67 in Control, BPH, and BPH + GSK-J4 groups, respectively.

To evaluate the therapeutic potential of GSK-J4 in androgen-driven benign prostatic hyperplasia (BPH), an in vivo study was conducted using C57BL/6 mice. The animals were assigned to three treatment groups for a duration of four weeks: vehicle control, testosterone, or testosterone co-administered with GSK-J4 (Fig. 8A). No statistically significant differences in body weight were observed among the groups (Fig. 8B). As expected, testosterone administration significantly elevated the prostate index in mice compared to the control group (*p* < 0.001). Co-treatment with GSK-J4 partially, but significantly, attenuated this testosterone-induced increase in prostate index (*p* < 0.05) (Fig. 8C). Histopathological analysis via HE staining revealed that testosterone treatment induced a pronounced inflammatory infiltrate within the prostate, reflected by a higher inflammation score. This inflammatory response was markedly reduced in mice receiving the combined testosterone and GSK-J4 regimen (Fig. 8D, E). Consistent with the histology, analysis of prostatic cytokine levels showed that testosterone significantly upregulated the expression of pro-inflammatory mediators, including IL-6, G-CSF, and MCP-1. This upregulation was significantly suppressed by GSK-J4 co-treatment (all *P* < 0.05) (Fig. S6A). In contrast, the expression levels of other cytokines, such as IL-10, IL-4, and CCL5, remained unchanged across the experimental groups (Fig. S6B–D). Immunohistochemical (IHC) staining further demonstrated that testosterone treatment significantly increased the protein expression of both JMJD3 and the proliferation marker Ki67 in prostatic tissues. Co-administration of GSK-J4 effectively counteracted these testosterone-induced increases in JMJD3 and Ki67 expression (all *P* <0.05) (Fig. 8F–H).

## Discussion

LUTS caused by BPH are a common diagnosis among the aging male population, with an increasing prevalence of 50–70% in men over 50 years of age and of up to 80% in men over 70 years of age [17]. Although the exact molecular pathological mechanisms causing BPH remain unclear, accumulated evidence revealed that inflammation plays a significant role in BPH progression as an acute mediator [18–20]. Alexandre et al. revealed that chronic inflammation was found in over 70% of male with BPH, and multivariate analyses showed that individuals with chronic inflammation have a higher BPH score than individuals without (*P* < 0.0001) [20]. Other well-designed and performed large clinical trials also indicate chronic inflammation in BPH as a potential cause for more severe lower urinary tract symptoms, larger prostate volumes, and a higher probability of acute urine retention [21, 22]. In this study, we also found significantly higher levels of inflammation in BPH tissues compared to normal prostate tissues, which is consistent with previous reports. In addition, our investigations showed that the expression of JMJD3 is significantly upregulated in BPH samples compared to normal samples. All of these findings, including our investigation, provide robust evidence supporting the significant roles of inflammation in the pathogenic mechanism of BPH.

Previously, a study revealed that inhibition of JMJD3 may down-regulate disease-relevant inflammatory responses in a H3K27-dependent manner [23]. To investigate the relationship between inflammation and JMJD3 expression, LPS was employed to make BPH models in vitro and in vivo. The results of this study showed that the effects of LPS inducing JMJD3 expression and inflammation responses are significantly inhibited by JMJD3 inhibitor GSK-J4. Then, Yu et al. found that the upregulated JMJD3 in osteoarthritis demethylates H3K27me3 at the NR4A1 promoter to promote its expression and promote the progression of osteoarthritis [11]. Another investigation indicated that JMJD3 directs early inflammation in nondiabetic wound macrophages via the JAK1/3/STAT3 pathway [24]. Furthermore, we employed plasmids to overexpress JMJD3 in RWPE-1 and WPMY-1 cells, respectively. And we found that overexpression of JMJD3 promotes cell proliferation and cell cycles in RWPE-1 and WPMY-1 cells, respectively. Nevertheless, GSK-J4, the inhibitor of JMJD3, could inhibit cell proliferation and cell cycles, and promote cell apoptosis in BPH-1 cells. Taken together, all the above evidence supported that JMJD3 plays a critical role in the progression of inflammation-related diseases, including BPH.

To investigate the mechanisms of JMJD3 regulating the progression of BPH, RNA sequencing was performed in RWPE-1 and WPMY-1 cells with JMJD3 overexpression, respectively. In this study, the results of RNA sequencing showed that pathways related to histone methylation are significantly downregulated, and the TGF-β pathway is significantly upregulated in RWPE-1 and WPMY-1 cells with JMJD3 overexpression, respectively. TGF-β, as a multifunctional cytokine, is known to drive diverse cellular responses, including epithelial-mesenchymal transition, embryonic development, tissue homeostasis, cellular immunity, proliferation, apoptosis, and cancer progression [25–28]. Previous studies demonstrated that TGF- β1 is highly expressed and activated in BPH and contributes to disease progression of BPH [29–32]. In this study, we illustrated that overexpressed JMJD3 demethylates H3K27me3 in the promotor of TGF-β1 to promote its expression and promote cells proliferation in BPH rather than TGF-β2 or TGF-β3. Aashaq et al., reported that TGF-β1 as a multifunctional cytokine activates downstream Smad2/3 through phosphorylation and is involved in regulating various cellular activities like proliferation, apoptosis, inflammation [31, 33]. Furthermore, a previous study found that there are a total of 497 differentially expressed genes in primary stromal prostate cells with and without TGF-β1 treatment, and pathway enrichment analyses of differentially expressed genes shows Wnt signaling pathway, PI3K−Akt signaling pathway, JAK − STAT signaling pathway, and Hippo signaling pathway activated [29]. Therefore, further studies will be designed to investigate the involvement of these indicated pathways in BPH progression.

Additionally, there are still some limitations in this study. Firstly, although we performed rescue assays to confirm that LPS promotes the progression of BPH via upregulating JMJD3 expression, the exact mechanisms of LPS upregulating JMJD3 expression remain unclear. Our findings support an association between JMJD3 activation and ROS production, but don’t definitively establish TGF-β1 as the sole mediator of JMJD3-induced ROS production. The precise molecular mechanism warrants further investigation. Secondly, we confirmed that overexpressing JMJD3 could activating TGF-β1 signaling in a H3K27me3-dependent manner; further studies are needed to investigate the exact binding sites of H3K27me3 in the promotor region of TGF- β1. Third, cell-type proportions derived from scRNA-seq represent recovered cells after tissue processing and filtering and therefore may not fully reflect the true histological composition of prostate tissue. Accordingly, our conclusions regarding KDM6B were based mainly on within-cell-type expression differences rather than inferred abundance shifts. Then, there are limited therapeutic strategies other than surgery for men with lower urinary tract symptoms associated with BPH, and the therapeutic options have not been updated markedly over the past decades. Finally, we revealed that small molecule GSK-J4, the inhibitor of JMJD3, shows promising effects on BPH management in cell and animal models, but both further experiments and clinical trials are still needed before GSK-J4 is employed for BPH patients’ management.

## Conclusions

In summary, our investigations indicated that JMJD3 is upregulated in BPH and is a critical factor in the progression of BPH. Therefore, targeting JMJD3 with GSK-J4 may be a promising strategy for BPH treatment.

## Supplementary information

Supplemental Material (Supplemental Figures and Tables)

Related Manuscript File (raw Gel Blot)
