# Supplementary material for: JMJD3 modulates benign prostatic hyperplasia through epigenetic regulation of TGF-β1
Source: Cell Death Dis. 2026 Jun 5;17(1):701. doi: 10.1038/s41419-026-08967-9 (PMC13454434; doi:10.1038/s41419-026-08967-9)

Figure 2

D

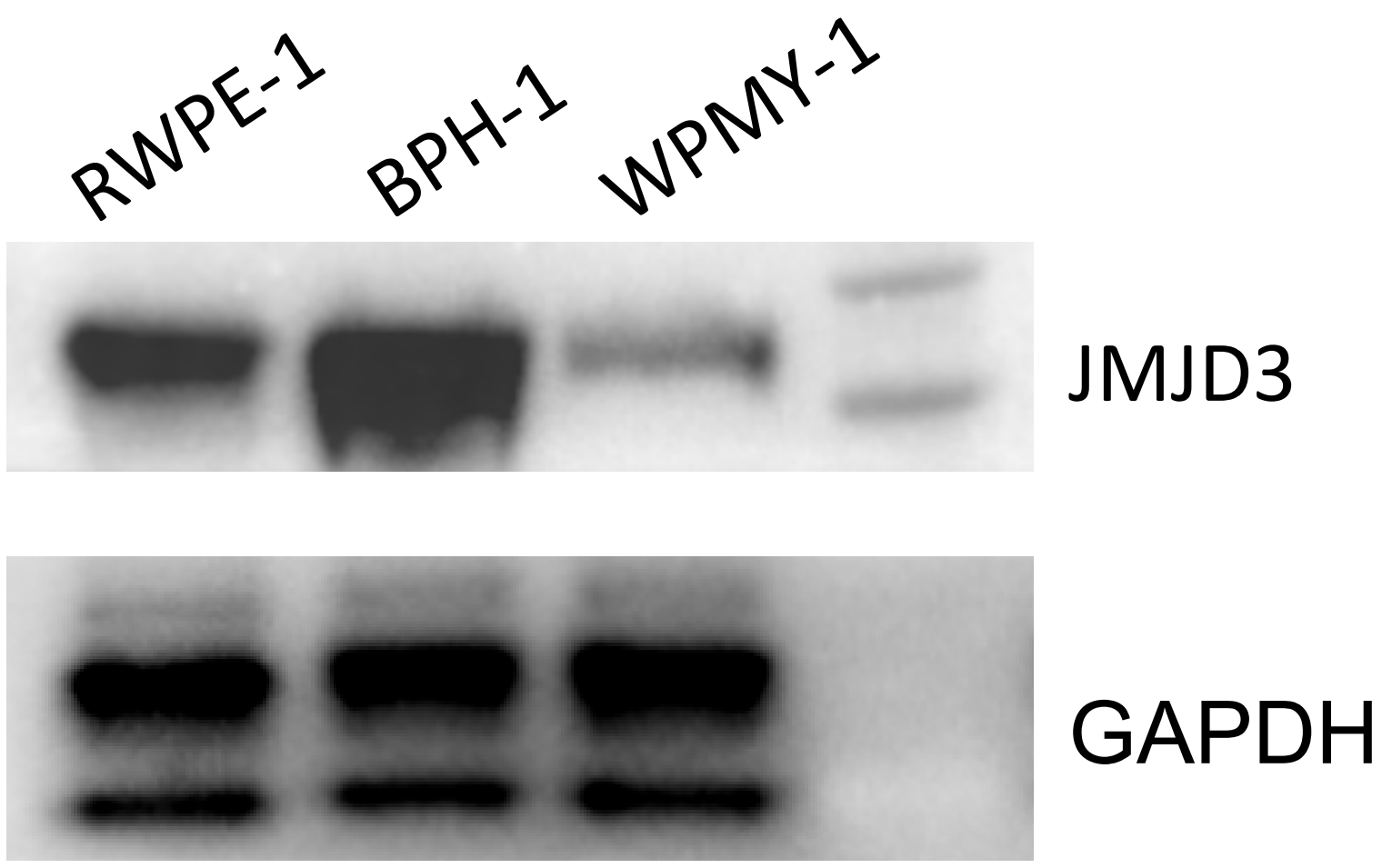
RWPE-1
BPH-1
WPMY-1
JMJD3
GAPDH

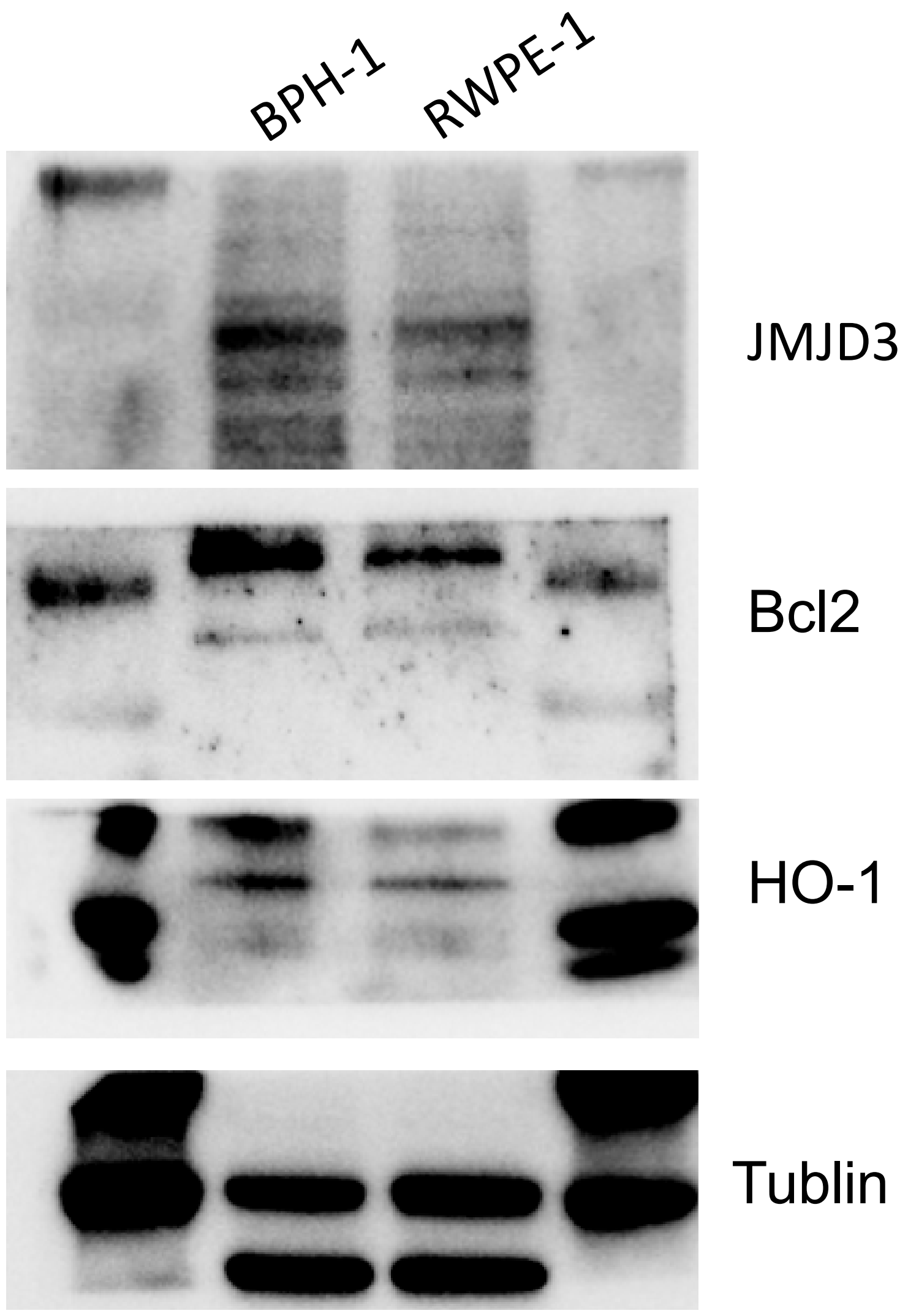
BPH-1
RWPE-1
JMJD3
Bcl2
HO-1
Tublin

Figure 3

B

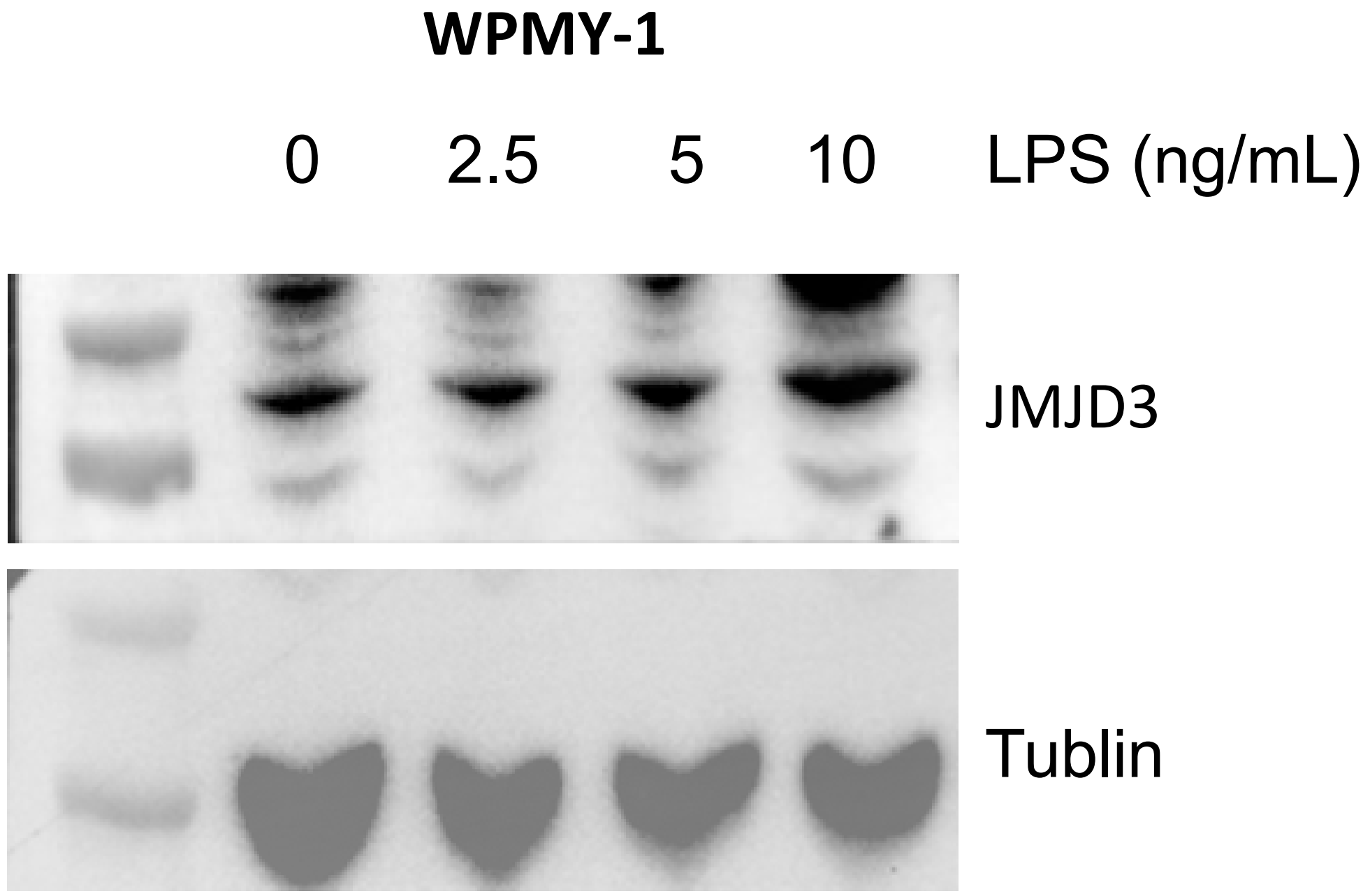

WPMY-1
0
2.5
5
10
LPS (ng/mL)
JMJD3
Tublin

C

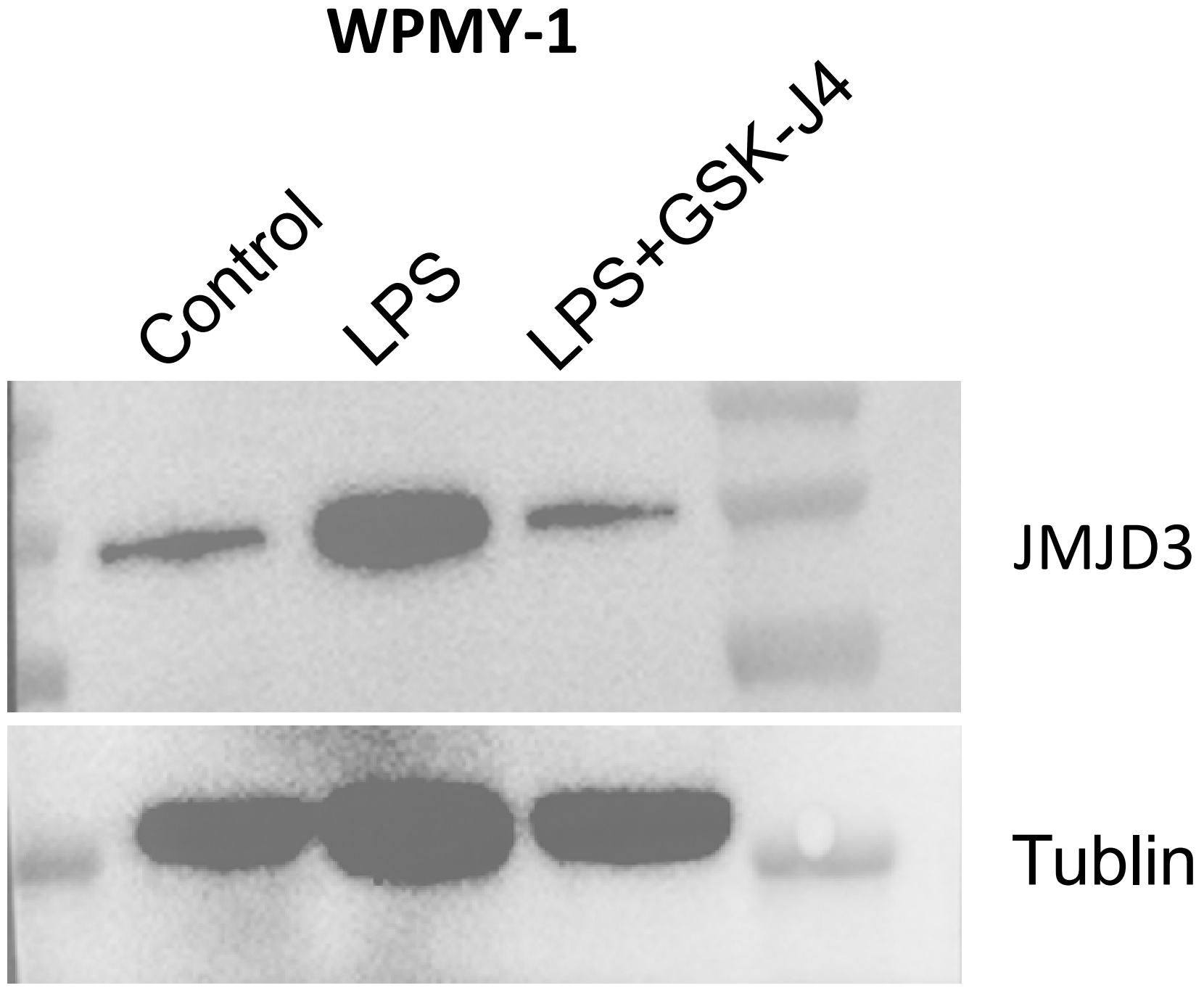

WPMY-1
Control
LPS
LPS+GSK-J4
JMJD3
Tublin

Figure 4

B

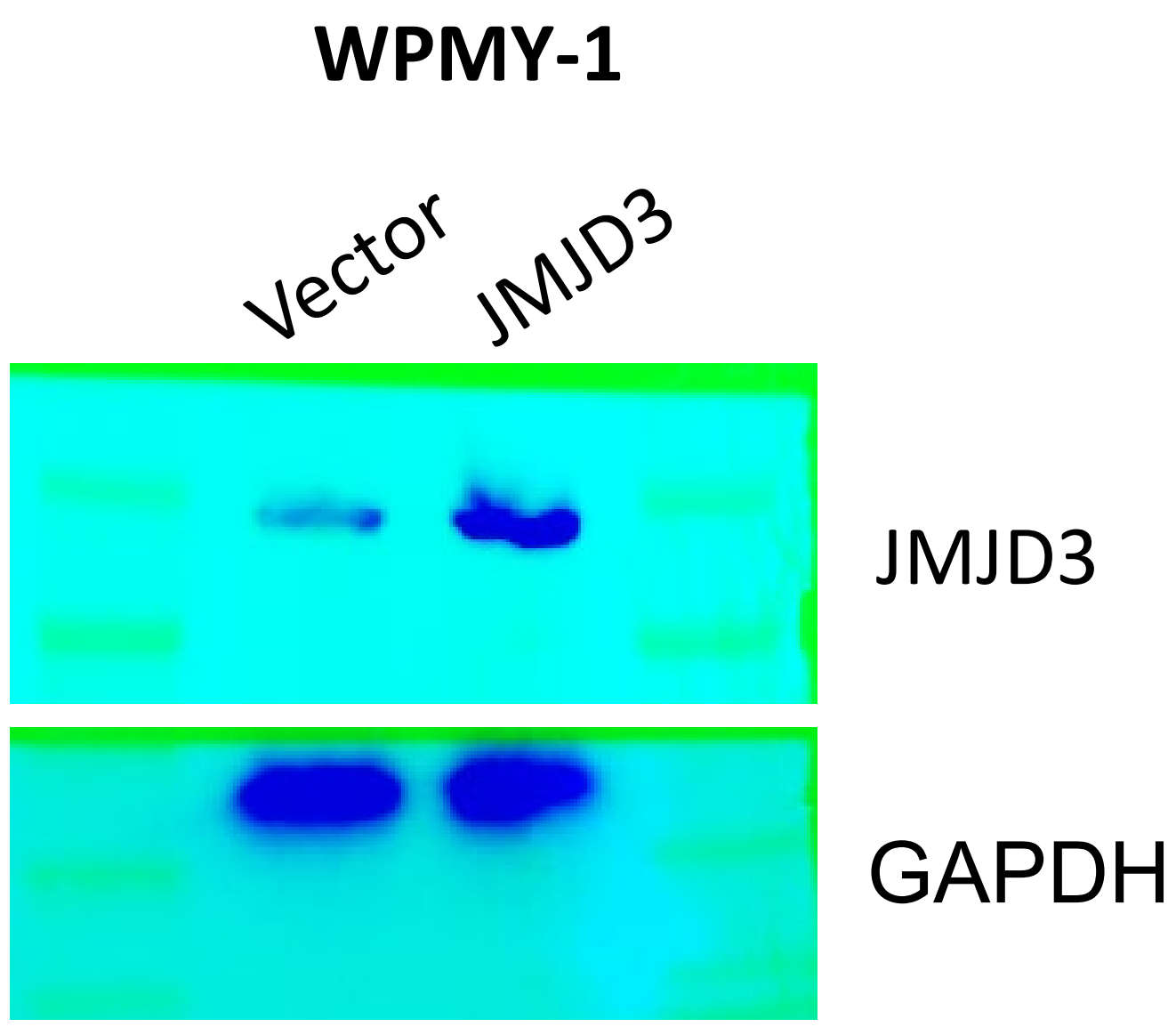

WPMY-1
Vector
JMJD3
JMJD3
GAPDH

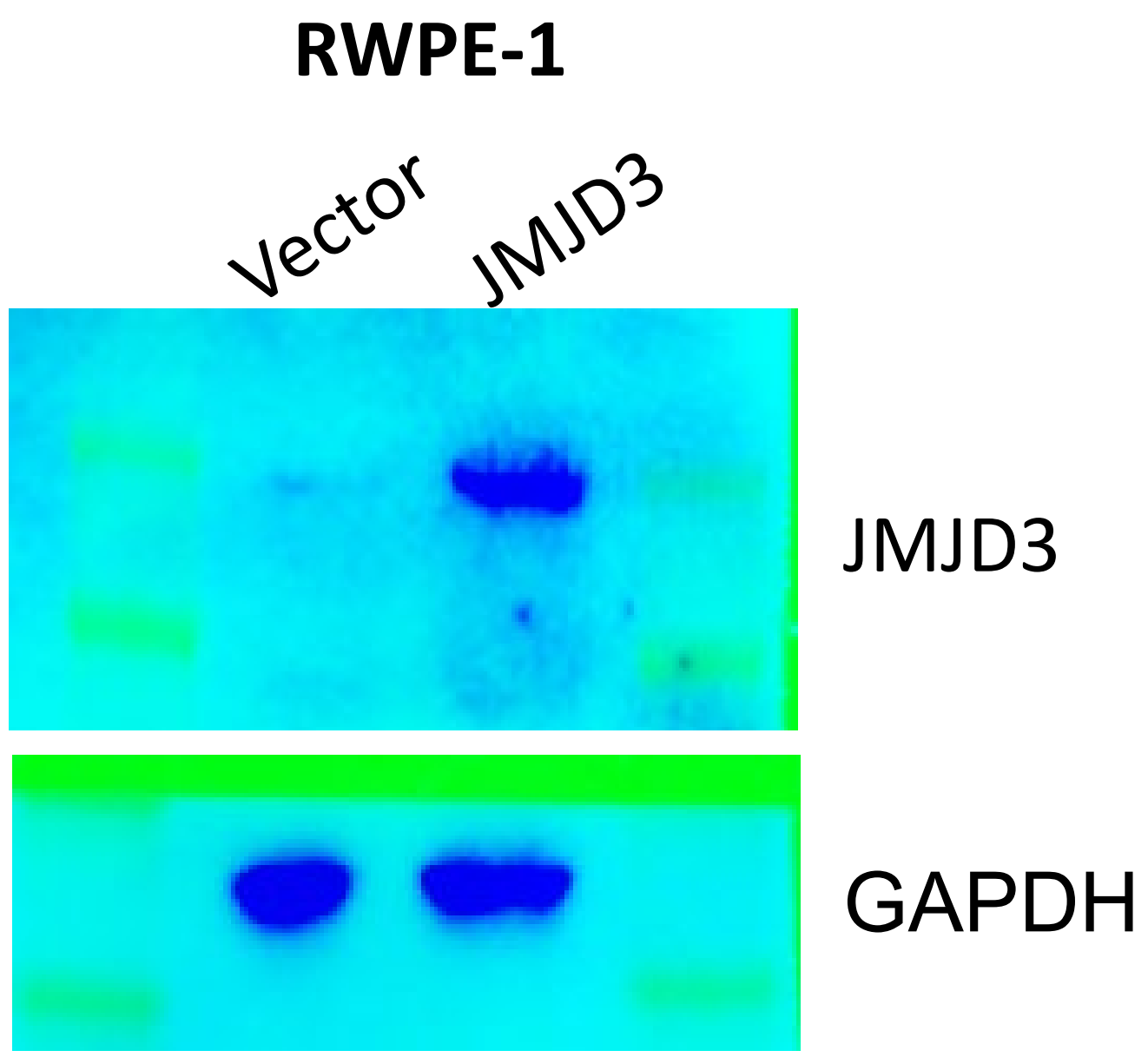

RWPE-1
Vector
JMJD3
JMJD3
GAPDH

Figure 7

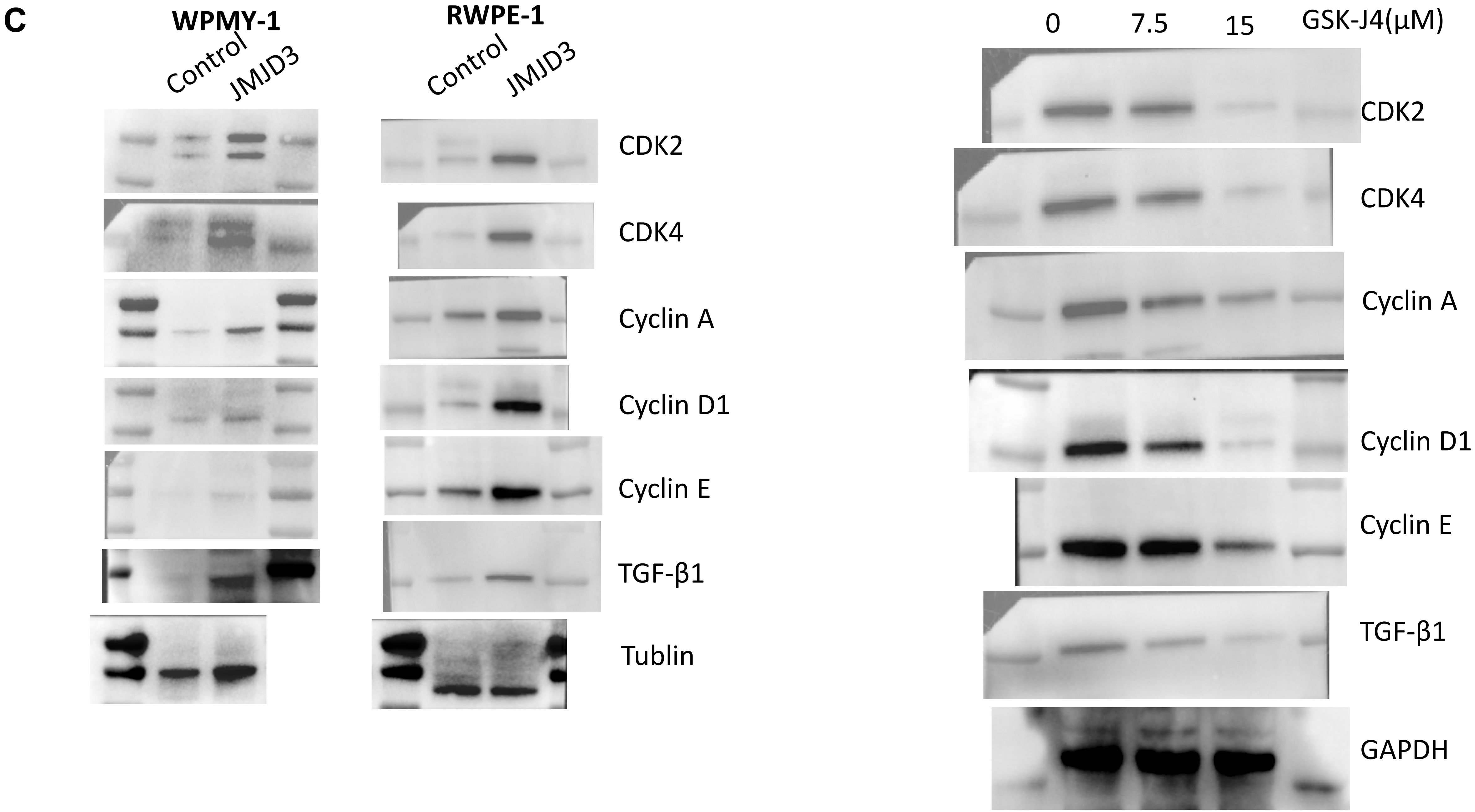
C
WPMY-1
Control
JMJD3
RWPE-1
Control
JMJD3
CDK2
CDK4
Cyclin A
Cyclin D1
Cyclin E
TGF-β1
Tublin
D
BPH-1
0
7.5
15
GSK-J4(μM)
CDK2
CDK4
Cyclin A
Cyclin D1
Cyclin E
TGF-β1
GAPDH

Figure 7

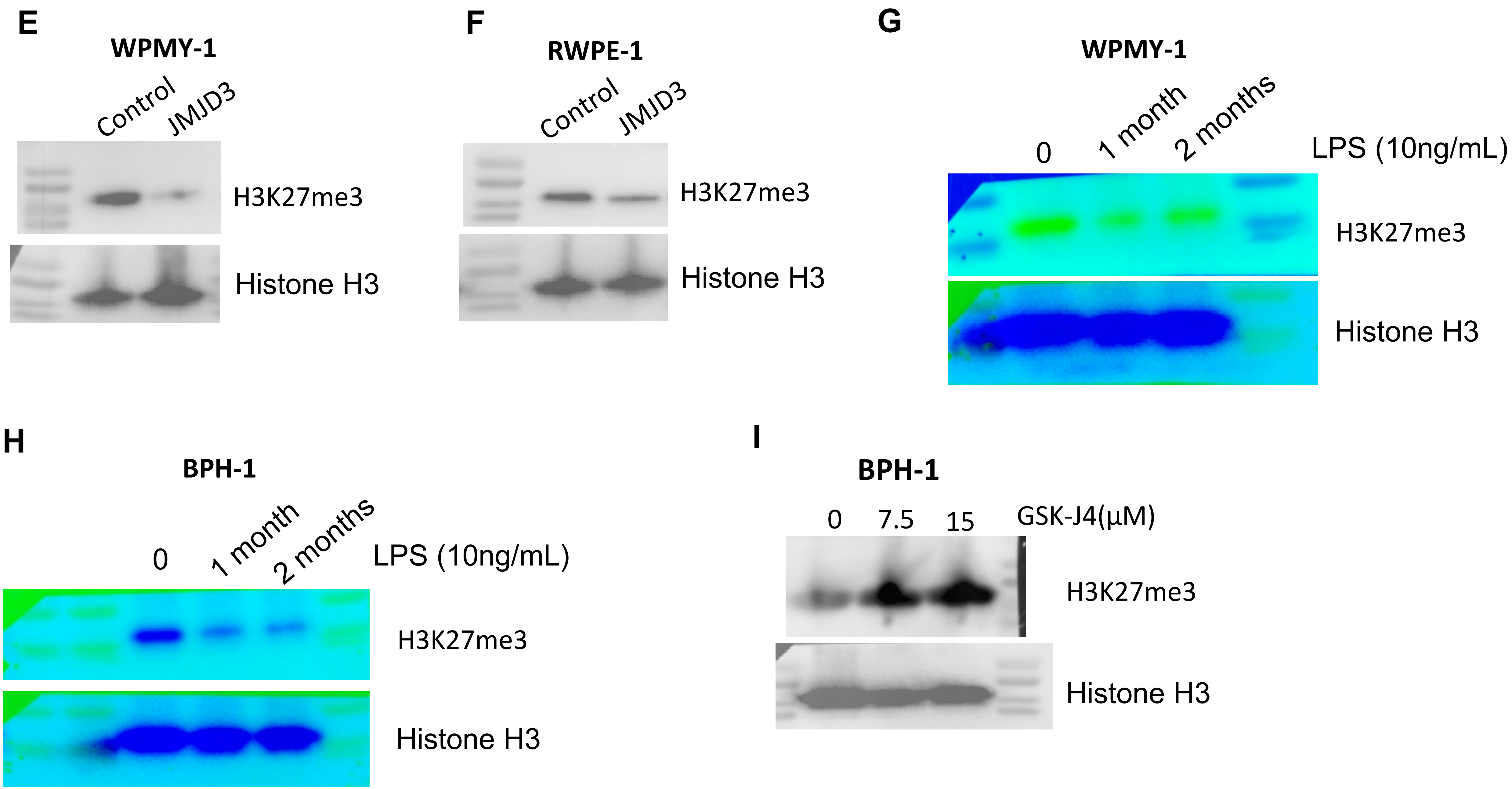
E
WPMY-1
Control
JMJD3
H3K27me3
Histone H3
F
RWPE-1
Control
JMJD3
H3K27me3
Histone H3
G
WPMY-1
0
1 month
2 months
LPS (10ng/mL)
H3K27me3
Histone H3
H
BPH-1
0
1 month
2 months
LPS (10ng/mL)
H3K27me3
Histone H3
I
BPH-1
0
7.5
15
GSK-J4(μM)
H3K27me3
Histone H3

S Figure 3

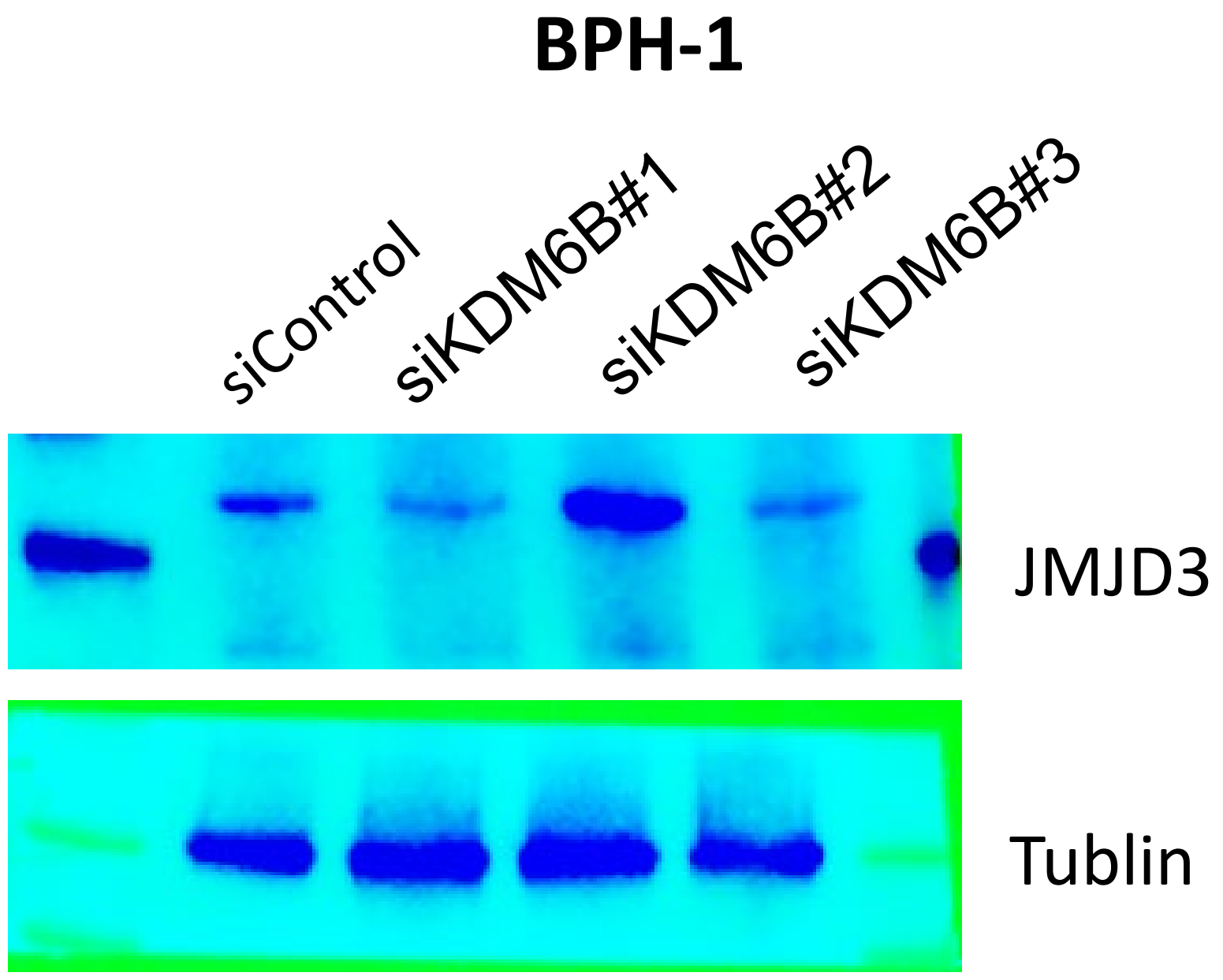

Supplement: Supplementary file 1 [file 41419_2026_8967_MOESM2_ESM.pdf]
