# Supplementary material for: JMJD3 modulates benign prostatic hyperplasia through epigenetic regulation of TGF-β1
Source: Cell Death Dis. 2026 Jun 5;17(1):701. doi: 10.1038/s41419-026-08967-9 (PMC13454434; doi:10.1038/s41419-026-08967-9)
Supplement: Supplementary file 2 [file 41419_2026_8967_MOESM1_ESM.docx]

**Supplementary Information for**

**JMJD3 modulates benign prostatic hyperplasia through epigenetic regulation of TGF-β1**

**Authors:** Bo Chen, Jinze Li, Yin Huang, Xinyang Liao, Mingjing He, Zeyu Chen, Jia You, Jie Chen, Qiyou Wu, Jinbao Wang, Jinjiang Jiang, Jingxing Bai, Biao Ran, Chunlan Chen, Shu Ning, Dehong Cao*, Qiang Wei*, Liangren Liu*

***Corresponding author:** Dehong Cao, Qiang Wei& Liangren Liu

**Email:** **[caodehong@scu.edu.cn](mailto:caodehong@scu.edu.cn); weiqiang@scu.edu.cn;** [**liuliangren@scu.edu.cn**](mailto:liuliangren@scu.edu.cn)

**This file includes:**

**Figures S1 to S6, and Table S1 to S3.**

**Supplemental Figure 1.** Single cell sequence reveals the features of KDM6B+cells and cell-cell communication networks.

**A**. tNSE and UMAP plots showed 23 cell clusters derived from single cell sequence data in normal and benign prostatic hyperplasia samples.

**B**. Inferred intercellular communication networks using CellChat. The left panel shows the number of ligand-receptor interactions between cell types, while the right panel indicates the interaction strength/weights.

**C**. Volcano plots displaying differentially expressed genes (|Log_2_Foldchange|≥0.5; *P*＜0.05) between KDM6B+ and KDM6B- cells. Comparisons were performed across all BPH cells, basal cells, luminal cells, and fibroblast cells from BPH samples, revealing extensive upregulated (red) and downregulated (blue) genes in KDM6B+ cells.

**D**. Pathway enrichment analysis of upregulated genes in KDM6B+ luminal cells from BPH samples.

**Supplemental Figure 2.** Immunofuorescence staining shows the expression and location of JMJD3 in WPMY-1 and BPH-1 cells.

**A-B**. Immunofuorescence staining of JMJD3 in WPMY-1 (**A**) and BPH-1 (**B**) cells

**Supplemental Figure 3.** KDM6B knockdown inhibits proliferation and induces apoptosis in BPH-1 cells.

(**A**) RT–PCR analysis of KDM6B mRNA expression in BPH-1 cells transfected with control siRNA (siControl) or three independent siRNAs targeting KDM6B (siKDM6B#1–#3) for 72 hours. Relative expression levels were normalized to internal controls. (**B**) Western blot analysis of JMJD3 (KDM6B) protein levels following siRNA-mediated knockdown in BPH-1 cells. Tubulin served as a loading control. (**C**) CCK-8 assay showing cell viability at 0, 24, and 48 hours after transfection. (**D**) Representative immunofluorescence images of EdU incorporation (red) in BPH-1 cells. Nuclei were counterstained with DAPI (blue). (**E**) Quantification of EdU-positive cells, expressed as the percentage of total nuclei. (**F**) Flow cytometric analysis of cell cycle distribution in BPH-1 cells after KDM6B silencing. Representative histograms (left) and quantification of G0/G1, S, and G2/M phase proportions (right) are shown. (**G**) Annexin V/PI staining followed by flow cytometry to assess apoptosis. Representative dot plots (left) and quantification of apoptotic rates (right) are shown.

**Supplemental Figure 4.** Overexpressing JMJD3 promotes genes reprogramming in WPMY-1 and RWPE-1 cells.

**A-B**. Enrichment plots of GSEA analyses for pathways in overexpressing JMJD3 group compared with vector group in WPMY-1 (**A**) and RWPE-1 (**B**) cells.

**Supplemental Figure 5.** CHIP sequencing demonstrates the effects of H3K27me3 epigenetically regulating TGF-β2 and TGF-β3 signaling.

**A**. CHIP sequencing of histone H3K27 trimethylation (H3K27me3) in GSE255550 dataset was performed with mouse bone marrow cells. And the plot showing the normalized H3K27-me3 peaks at TGF-β2 and TGF-β3.

**B**. CHIP sequencing of H3K27me3 in GSE138157 dataset was performed with primary liver cells of C57BL/6 mouse. And the plot showing the normalized H3K27-me3 peaks at TGF-β2 and TGF-β3.

**Supplemental Figure 6.** The effects of GSK-J4 on regulating the protein levels related to inflammation response in serum of mice.

**A**. Luminex liquid suspension chip assay was performed to determine the protein levels of IL-6, G-CSF, MIP-1β, IL-5, and MCP-1 in Control, BPH, and BPH+GSK-J4 group.

**B**. Luminex liquid suspension chip assay was used to test the protein levels of GM-CSF, KC, IL-10, TNF-α, IL-2, and IL-3 in Control, BPH, and BPH+GSK-J4 group.

**C**. Luminex liquid suspension chip assay was performed to determine the protein levels of MIP-1 α, IL-1α, IL-1β, IL-4, and IL-13 in Control, BPH, and BPH+GSK-J4 group.

**D**. Luminex liquid suspension chip assay was used to test the protein levels of IL-12 (p40), CCL5, IL-12 (p70), IL-9 and IFN-γ in Control, BPH, and BPH+GSK-J4 group.

**Table S1. The sequences of siRNA for targeting *KDM6B*.**

| **Target** | **Number** | **Direction** | **Sequence** |
| --- | --- | --- | --- |
|  | siControl | Sense | UUCUCCGAACGUGUCACGUdTdT |
|  |  | Antisense | ACGUGACACGUUCGGAGAAdTdT |
|  | siKDM6B#1 | Sense | GCUACACCUUGAGCACAAAdTdT |
| *KDM6B* |  | Antisense | UUUGUGCUCAAGGUGUAGCdTdT |
|  | siKDM6B#2 | Sense | GGAAUGAGGUGAAGAACGUdTdT |
|  |  | Antisense | ACGUUCUUCACCUCAUUCCdTdT |
|  | siKDM6B#3 | Sense | GCAUCUAUCUGGAGAGCAAdTdT |
|  |  | Antisense | UUGCUCUCCAGAUAGAUGCdTdT |

**Table S2.** The primer sequences for RT-PCR.

| **Species** | **Gene symbol** | **Direction** | **Sequence (5'->3')** |
| --- | --- | --- | --- |
| Human | *KDM1A* | Forward | GTGGACGAGTTGCCACATTTC |
|  |  | Reverse | TGACCACAGCCATAGGATTCC |
| Human | *KDM4C* | Forward | CGAGGTGGAAAGTCCTCTGAA |
|  |  | Reverse | GGGCTCCTTTAGACTCCATGTAT |
| Human | *KDM5B* | Forward | CCATAGCCGAGCAGACTGG |
|  |  | Reverse | GGATACGTGGCGTAAAATGAAGT |
| Human | *PHF2* | Forward | CTCCCCTACGACGTTACCC |
|  |  | Reverse | CAGTGGTATATGTCGATGTCGG |
| Human | *PHF8* | Forward | GTGCCGGTGTATTGCCTCT |
|  |  | Reverse | CAACACAACTGCCATGAAACC |
| Human | *JARID2* | Forward | ACCAGTCTAAGGGATTAGGACC |
|  |  | Reverse | TGCTGGGACTATTCGGCTGA |
| Human | *JMJD6* | Forward | GGTGAGGATAACGATGGCTACT |
|  |  | Reverse | GCTGTCAAAGATGTAAAGGGGAC |
| Human | *JMJD7* | Forward | CGGCAACCTACCAGCTAACTG |
|  |  | Reverse | GGGCCTGACTGTAACTAGGGT |
| Human | *KDM8* | Forward | GGCCCGTGATCCTGAAAGG |
|  |  | Reverse | ACCTCGAACCAACTTCCACTG |
| Human | *UTY* | Forward | CGCAGTGTCGCTCACTACC |
|  |  | Reverse | GTCAGGCTAACAGACTCCTCTT |
| Human | *JMJD4* | Forward | GTCTGTCAATGTCTGTGGGAG |
|  |  | Reverse | CAGGTGTGTGTCGCAGAGT |
| Human | *KDM1B* | Forward | CTCTCCTGTGGGGAACATTTC |
|  |  | Reverse | GACTAGGTTCGGTTTTGCCATT |
| Human | *KDM5C* | Forward | GGGTCCGACGATTTCCTACC |
|  |  | Reverse | ATGCCCGATTTCTCTGCGATG |
| Human | *KDM6A* | Forward | TACAGGCTCAGTTGTGTAACCT |
|  |  | Reverse | CTGCGGGAATTGGTAGGCTC |
| Human | *KDM7A* | Forward | TGCTGTGCTCACTTCTCAGG |
|  |  | Reverse | CAAAGAAACGTGCATGGCCA |
| Human | *JMJD8* | Forward | AGTGGGCCTCTCTCTTTCG |
|  |  | Reverse | CCAGCTCCTGCGATTCCAAA |
| Human | *KDM2A* | Forward | CCAAAGGTGCGGGTTCCTAC |
|  |  | Reverse | GGCTCCTGACACAATCGGG |
| Human | *KDM2B* | Forward | TGTCCCAGTTTGTGCGTTACT |
|  |  | Reverse | GGACGCTTGACCAAGTGCT |
| Human | *KDM4A* | Forward | ATCCCAGTGCTAGGATAATGACC |
|  |  | Reverse | ACTCTTTTGGAGGAACAACCTTG |
| Human | *KDM9* | Forward | CCAGTACCTACCTCGGACCA |
|  |  | Reverse | GTACGCACTGGGAAACTGGA |
| Human | *JMJD1C* | Forward | CAGGTCTCGTGCCAATCAAAA |
|  |  | Reverse | GCTGTTGCTGGTGTGTATTCT |
| Human | *KDM3B* | Forward | CCCCAGGACCTAGCGATCTT |
|  |  | Reverse | AGCAGAAGCACGATAACTTCC |
| Human | *KDM4B* | Forward | ACTTCAACAAATACGTGGCCTAC |
|  |  | Reverse | CGATGTCATCATACGTCTGCC |
| Human | *KDM5A* | Forward | AGCCGAGTTGGGAGGAGTT |
|  |  | Reverse | TGGACTCTTGGAGTGAAACGA |
| Human | *KDM6B* | Forward | CACCCCAGCAAACCATATTATGC |
|  |  | Reverse | CACACAGCCATGCAGGGATT |
| Human | *GAPDH* | Forward | ATGAGTCCTTCCACGATACCA |
|  |  | Reverse | GAAATCCCATCACCATCTTCC |

**Table S3.** The details of primary antibodies.

| **Antibodies** | **Dilution** | **Company** | **Cat#** |
| --- | --- | --- | --- |
| JMJD3 | 1:1000 (WB)  1:100 (IHC or IF) | ABclonal | A12763 |
| BCL-2 | 1:2000 | Proteintech | 12789-1-AP |
| HO-1 | 1:5000 | Proteintech | 10701-1-AP |
| CDK2 | 1:1000 | Zenbio | R22532 |
| CDK4 | 1:1000 | Zenbio | R23888 |
| CyclinA | 1:1000 | Zenbio | R27473 |
| CyclinD1 | 1:1000 | Zenbio | R380999 |
| CyclinE | 1:1000 | Zenbio | R381029 |
| TGF-β1 | 1:1000 | ABclonal | A2124 |
| H3K27me3 | 1:1000 | Cell Signaling Technology | 9733S |
| H3 | 1:1000 | Zenbio | 250182 |
| Ki67 | 1:100 (IHC) | Zenbio | R10018 |
| GAPDH | 1:1000 | Cell Signaling Technology | 2118S |
| Tublin | 1:5000 | Zenbio | T200608 |
| Actin | 1:1000 | Cell Signaling Technology | 4967S |
